# Supplementary material for: Natural Products of Marine Macroalgae from South Eastern Australia, with Emphasis on the Port Phillip Bay and Heads Regions of Victoria
Source: Mar Drugs. 2020 Feb 28;18(3):142. doi: 10.3390/md18030142 (PMC7143075; doi:10.3390/md18030142)
Supplement: Supplementary file 1 [file marinedrugs-18-00142-s001.pdf]

Natural Products of Marine Macroalgae from South Eastern Australia, with Emphasis on the Port Phillip Bay and Heads Regions of Victoria

James Lever[1], Robert Brkljača[1, 2], Gerald Kraft[3, 4] and Sylvia Urban[1*]

[1] School of Science (Applied Chemistry and Environmental Science), RMIT University, GPO Box 2476V Melbourne, VIC 3001, Australia.

[2] Monash Biomedical Imaging, Monash University, Clayton, VIC, Australia.

[3] School of Biosciences, University of Melbourne, Parkville, Victoria 3010, Australia.

[4] Tasmania Herbarium, College Road, Sandy Bay, Tasmania 7015, Australia.

Supporting Information

S1. Taxonomic Revisions.

S2. Diterpenes Isolated from Green Algae.

S3. Sesquiterpenes Isolated from Green Algae.

S4. Cyclic Geranyl Acetones Isolated from Green Algae.

S5. Steroids/Sterols Isolated from Green Algae.

S6. Miscellaneous Compounds Isolated from Green Algae.

S7. Biological Activity Summary of Green Algae (Chlorophyta).

S8. Tocotrienols Isolated from Brown Algae.

S9. Monoterpenes Isolated from Brown Algae.

S10. Prenylated Phenols Isolated from Brown Algae.

S11. Merioditerpenoids Isolated from Brown Algae.

S12. Sesquiterpenes and Monoterpenes Isolated from Brown Algae.

S13. Diterpenoids Isolated from Brown Algae.

S14. Diterpenoids Isolated from Brown Algae (continued).

S15. Diterpenoids Isolated from Brown Algae (continued).

S16. Steroids/sterols Isolated from Brown Algae.

S17. Lipids and Polyenes Isolated from Brown Algae.

S18. Oxylipids and Epoxylipids Isolated from Brown Algae.

S19. Phloroglucinols Isolated from Brown Algae.

S20. Phenols/phenolic acids/resorcinols Isolated from Brown Algae.

S21. Miscellaneous Compounds Isolated from Brown Algae.

S22. Biological Activity Summary of Brown Algae (Ochrophyta).

S23. Halogenated Monoterpenes and Parguerenes Isolated from Red Algae.

S24. Chamigrenes Isolated from Red Algae.

S25. Laurenes Isolated from Red Algae.

S26. Sesquiterpenes Isolated from Red Algae

S27. Lauroxocanes Isolated from Red Algae

S28. Polyhalogenated Indoles Isolated from Red Algae.

S29. Polyhalogenated Hydrocarbons Isolated from Red Algae.

S30. Halogenated Furanones Isolated from Red Algae.

S31. Steroids Isolated from Red Algae.

S32. Miscellaneous Classes of Compounds Isolated from Red Algae.

S33. Biological Activity Summary of Red Algae (Rhodophyta).

---

*Corresponding author. Tel: +61 3 9925 3376
E-mail address: sylvia.urban@rmit.edu.au (S. Urban

S1. Taxonomic Revisions

Previous taxonomy	Revised taxonomy
Sargassum decipiens	*Phyllotricha decipiens*
Grateloupia filicina	*Grateloupia subpectinata*
Laurencia elata	*Corynecladia elata*

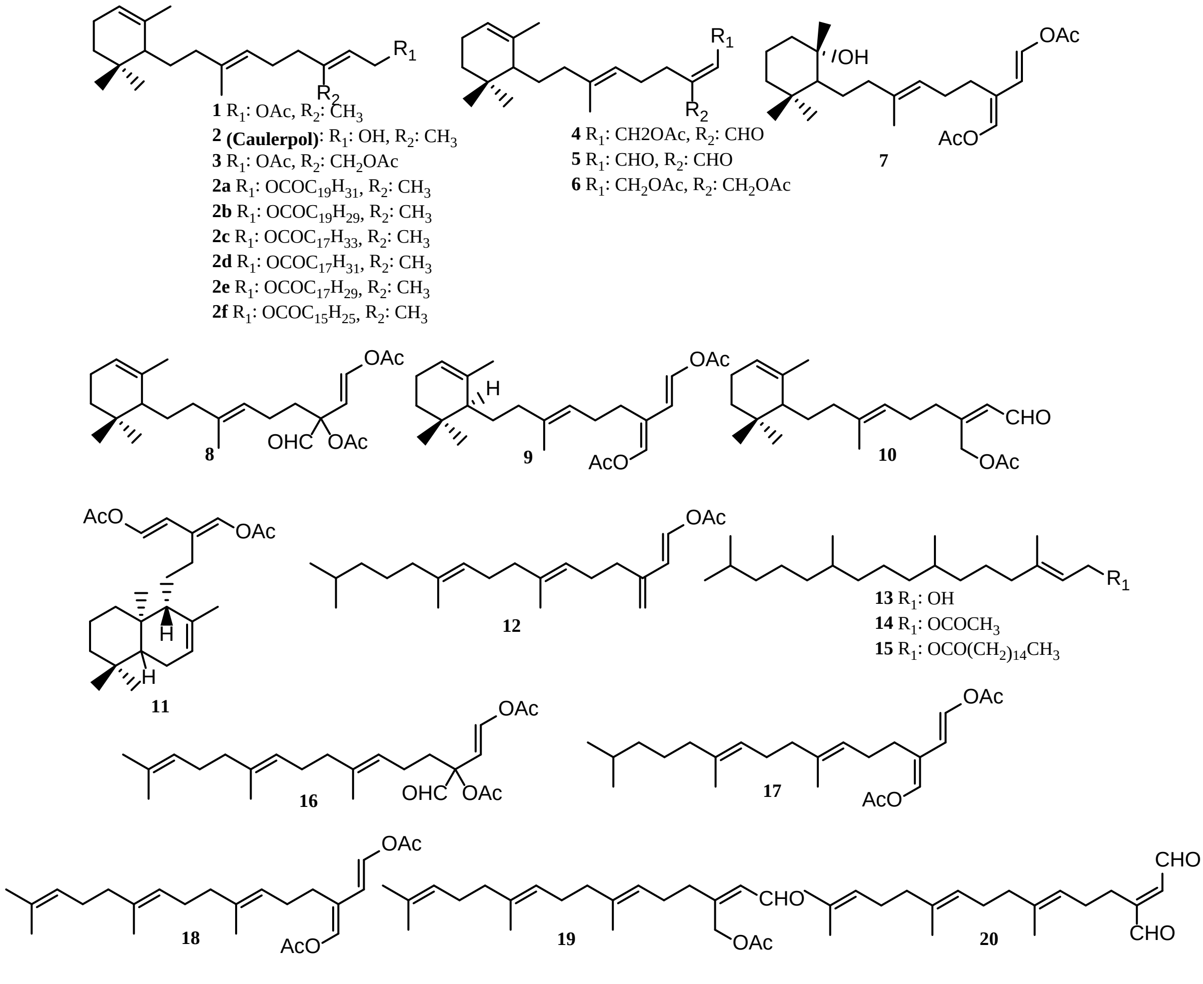

S2. Diterpenes Isolated from Green Algae.

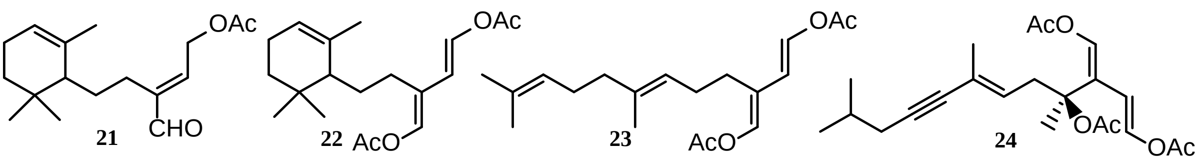

21 22 23 24

S3. Sesquiterpenes Isolated from Green Algae.

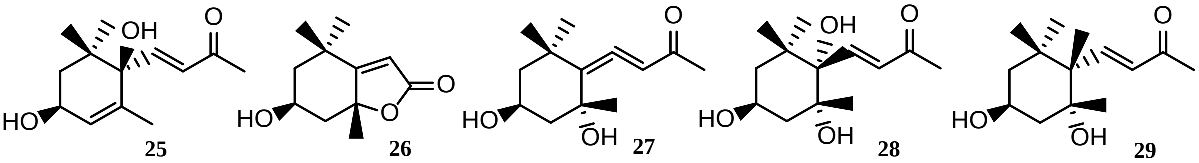

25 26 27 28 29

S4. Cyclic Geranyl Acetones Isolated from Green Algae.

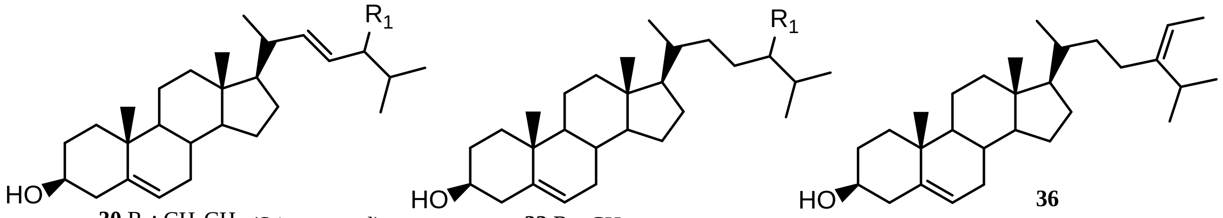

30 R_1: CH_2CH_3 (Stigmasterol)
31 R_1: H
32 R_1: CH_3 (Brassicasterol)

33 R_1: CH_3
34 R_1: CH_2CH_3 (b-sitosterol)
35 R_1: H (Cholesterol)

36

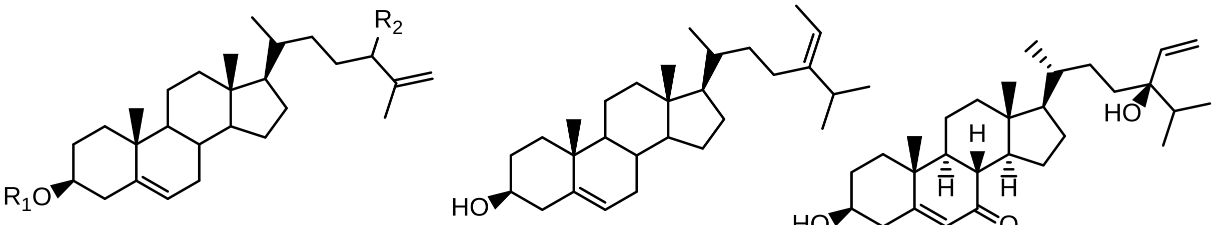

37 R_1: Ac, R_2: CH_3
38 R_1: $CO(CH_2)_{14}CH_3$, R_2: CH_3
39 R_1: H, R_2: CH_2CH_3 (Clerosterol)

40

41

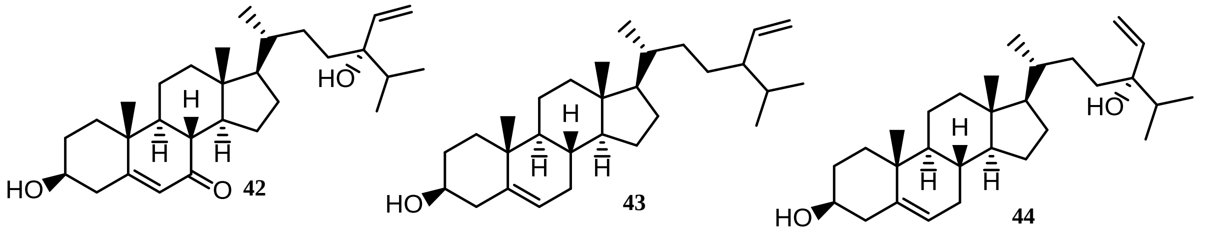

42 **43** **44**

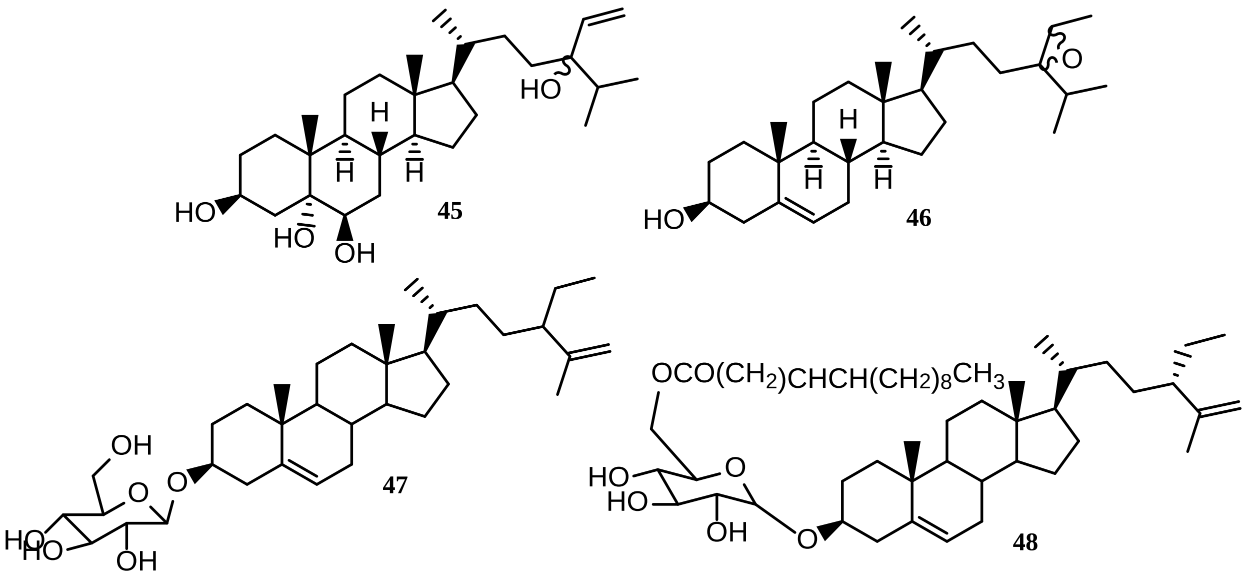

S5. Steroids/Sterols Isolated from Green Algae.

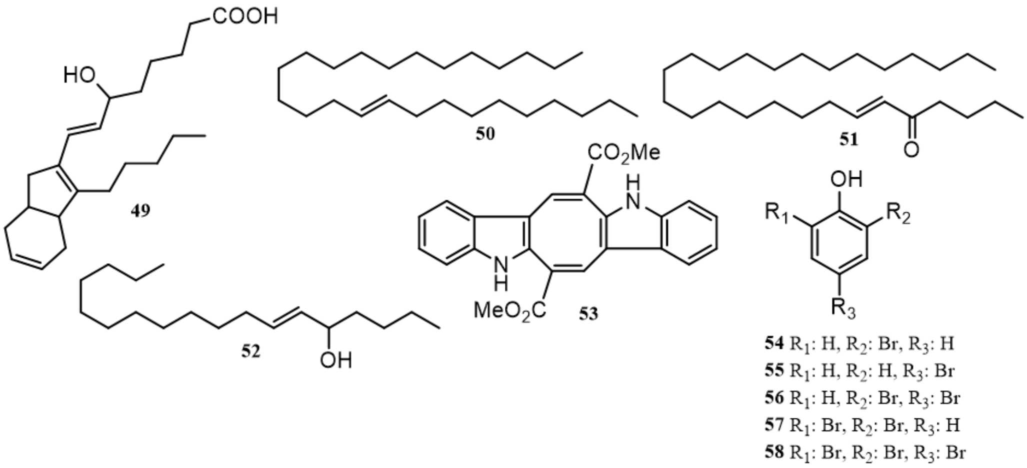

S6. Miscellaneous Compounds Isolated from Green Algae.

S7. Biological Activity Summary of Green Algae (Chlorophyta).

Species	Extract/Pure	Activity	Assay	Potency [Ref]
C. brownii	Pure Compound:			
		Antibacterial	*S. aureus* (G-)	>4 mm at 100 µg/disk [28]
	**7**	Antibacterial	*B. subtilus* (G+)	>4 mm at 100 µg/disk [28]
		Antibacterial	*E. coli* (G-)	>4 mm at 100 µg/disk [28]
		Antibacterial	*V. anguillarum* (G-)	>4 mm at 100 µg/disk [28]
C. flexilis	Pure Compound:			
		Antimicrobial	*B. subtilus* (G+)	>4 mm at 100 µg/disk [28]
	**23**	Antifungal	*D. haloides*	>4 mm at 100 µg/disk [28]
		Antifungal	*Lulworthia sp.*	>4 mm at 100 µg/disk [28]
		Cytotoxic	Fertilised sea urchin eggs	~ [28, 188]
C. trifaria	Pure Compound:			
	**5**	Cytotoxic	Brine Shrimp Assay	70% mortality at 10 mg/mL, 24 h [22]
	**9**	Cytotoxic	Brine Shrimp Assay	40% mortality at 10 mg/mL, 24 h [22]
		Antimicrobial	*B. subtilus* (G+)	>4 mm at 100 µg/disk [28]
	**23**	Antifungal	*D. haloides*	>4 mm at 100 µg/disk [28]
		Antifungal	*Lulworthia sp.*	>4 mm at 100 µg/disk [28]
		Cytotoxic	Fertilised sea urchin eggs	~ [28, 188]
C. scalpelliformis	Crude Extract:			
		Antibacterial	*K. pneumoniae* (G-)	6.2 mm at 25 µg/disk [189]
	Hexane	Antibacterial	*E. coli* (G-)	4.8 mm at 25 µg/disk [189]
		Antibacterial	*S. aureus* (G-)	6.0 mm at 25 µg/disk [189]
		Antibacterial	*P. aeruginosa* (G-)	1.4 mm at 25 µg/disk [189]
		Antibacterial	*E. aerogenes* (G-)	3.2 mm at 25 µg/disk [189]
		Antibacterial	*K. pneumoniae* (G-)	4.0 mm at 25 µg/disk [189]
	Chloroform	Antibacterial	*E. coli* (G-)	2.6 mm at 25 µg/disk [189]
		Antibacterial	*S. aureus* (G+)	3.4 mm at 25 µg/disk [189]
		Antibacterial	*P.aeruginosa* (G-)	3.6 mm at 25 µg/disk [189]
		Antibacterial	*E. aerogenes* (G-)	2.6 mm at 25 µg/disk [189]
		Antibacterial	*K. pneumoniae* (G-)	3.2 mm at 25 µg/disk [189]
	Ethyl acetate	Antibacterial	*E. coli* (G-)	2.2 mm at 25 µg/disk [189]
		Antibacterial	*S. aureus* (G+)	3.0 mm at 25 µg/disk [189]
		Antibacterial	*P.aeruginosa* (G-)	4.0 mm at 25 µg/disk [189]
		Antibacterial	*B. subtilus* (G+)	5.4 mm at 25 µg/disk [189]
	Acetone	Antibacterial	*E. aerogenes* (G-)	14.0 mm at 25 µg/disk [189]
		Antibacterial	*K. pneumoniae* (G-)	8.8 mm at 25 µg/disk [189]
		Antibacterial	*E. coli* (G-)	8.0 mm at 25 µg/disk [189]
		Antibacterial	*S. aureus* (G+)	2.4 mm at 25 µg/disk [189]
		Antibacterial	*P.aeruginosa* (G-)	5.4 mm at 25 µg/disk [189]
		Antibacterial	*E. aerogenes* (G-)	2.6 mm at 25 µg/disk [189]
		Antibacterial	*K. pneumoniae* (G-)	2.0 mm at 25 µg/disk [189]
	Ethanol	Antibacterial	*E. coli* (G-)	2.2 mm at 25 µg/disk [189]
		Antibacterial	*S. aureus* (G+)	2.2 mm at 25 µg/disk [189]
		Antibacterial	*P.aeruginosa* (G-)	2.6 mm at 25 µg/disk [189]
		Antibacterial	*B. subtilus* (G+)	1.6 mm at 25 µg/disk [189]
		Antibacterial	*E. aerogenes* (G-)	4.0 mm at 25 µg/disk [189]
	Methanol	Antibacterial	*K. pneumoniae* (G-)	6.0 mm at 25 µg/disk [189]
		Antibacterial	*E. coli* (G-)	4.2 mm at 25 µg/disk [189]
		Antibacterial	*S. aureus* (G+)	5.2 mm at 25 µg/disk [189]
		Antibacterial	*P.aeruginosa* (G-)	5.6 mm at 25 µg/disk [189]

S3. Biological Activity Summary of Green Algae (Chlorophyta) (continued)				
C. duthieae	Crude Extract:			
	Methanol:Water 1:1	Antioxidant	DPPH scavenging	47.7% at 1 mg/mL [190]
		Antialzheimers	AChE inhibitor	IC_{50}: 0.14 mg/mL [190]
C. fragile	Pure Compound:			
	Clerosterol (**39**)	Antitumour	Human melanoma cells (A2058)	IC_{50}: 150 μM [191]
	Dimethylsulfonio propionate	Antipredation	Reduced grazing of *Strongylocentrotus droebachiensis*	~ [192]
	Crude Extracts:			
U. australis	Pure Compounds:			
	**41**	Antidiabetes	Recombinant aldose reductase inhibitors	3.31% at 3 μg/mL [36]
	**42**	Antidiabetes		4.08% at 3 μg/mL [36]
	**45**	Antidiabetes		2.87% at 3 μg/mL [36]
	**47**	Antidiabetes		8.13% at 3 μg/mL [36]
	**46**	Antidiabetes		31.28% at 3 μg/mL [36]
U. compressa	Crude Extracts:			
	Dichloromethane	Antioxidant	DPPH scavenging	IC_{50}: 453.3 μg/mL [194]
U. lactuca	Crude Extracts:			
	Methanol	Antibacterial	10 *S. aureus* strains USA100, USA 200, USA 300, USA 400, USA 500, USA 600, COL, Newman, SH 1000, *S. epidermis* (All G+)	Dependant on lunar cycle of algae collection [195]
	Methanol	Antibacterial	*S. aureus* (G+)	11 mm at 25 mg/mL [196]
		Antibacterial	*B. subtilis* (G+)	11 mm at 25 mg/mL [196]
		Antibacterial	*E. coli* (G-)	12 mm at 25 mg/mL [196]
		Antibacterial	*P. aeruginosa* (G-)	14 mm at 25 mg/mL [196]
		Antibacterial	*B. spp* (G+)	13 mm at 25 mg/mL [196]
		Antibacterial	*S. typhi* (G-)	13 mm at 25 mg/mL [196]
		Antibacterial	*S. epidermis* (G+)	11 mm at 25 mg/mL [196]
		Antibacterial	*K. spp* (G-)	14 mm at 25 mg/mL [196]
	Aqueous	Antibacterial	*S. aureus* (G+)	12 mm at 25 mg/mL [196]
		Antibacterial	*B. subtilis* (G+)	12 mm at 25 mg/mL [196]
		Antibacterial	*E. coli* (G-)	12 mm at 25 mg/mL [196]
		Antibacterial	*P. aeruginosa* (G-)	ND [196]
		Antibacterial	*B. spp* (G+)	13 mm at 25 mg/mL [196]
		Antibacterial	*S. typhi* (G-)	11 mm at 25 mg/mL [196]
		Antibacterial	*S. epidermis* (G+)	12 mm at 25 mg/mL [196]
		Antibacterial	*K. spp* (G-)	11 mm at 25 mg/mL [196]
	70% Acetone	Antibacterial	*B. subtilis* (G+)	19 mm at 100 mg/mL [197]
		Antibacterial	*Staph. aereus* (G+)	17 mm at 100 mg/mL [197]
		Antibacterial	*Strept. aereus* (G+)	16 mm at 100 mg/mL [197]
		Antibacterial	*E. coli* (G-)	16 mm at 100 mg/mL [197]
		Antibacterial	*S. typhi* (G-)	17 mm at 100 mg/mL [197]
		Antibacterial	*K. pneumoniae* (G-)	16.5 mm at 100 mg/mL [197]
		Antifungal	*C. albicans*	16 mm at 100 mg/mL [197]
	70% Methanol	Antibacterial	*B. subtilis* (G+)	16 mm at 100 mg/mL [197]
		Antibacterial	*Staph. aereus* (G+)	12 mm at 100 mg/mL [197]
		Antibacterial	*Strept. aereus* (G+)	13 mm at 100 mg/mL [197]
		Antibacterial	*E. coli* (G-)	13 mm at 100 mg/mL[197]
		Antibacterial	*S. typhi* (G-)	13 mm at 100 mg/mL [197]
		Antibacterial	*K. pneumoniae* (G-)	16 mm at 100 mg/mL [197]
		Antifungal	*C. albicans*	15 mm at 100 mg/mL [197]
	70% Ethanol	Antibacterial	*B. subtilis* (G+)	14 mm at 100 mg/mL [197]
		Antibacterial	*Staph. aereus* (G+)	12 mm at 100 mg/mL [197]
		Antibacterial	*Strept. aereus* (G+)	13 mm at 100 mg/mL [197]
		Antibacterial	*E. coli* (G-)	13 mm at 100 mg/mL [197]
		Antibacterial	*S. typhi* (G-)	13 mm at 100 mg/mL [197]
		Antibacterial	*K. pneumoniae* (G-)	14 mm at 100 mg/mL [197]
		Antifungal	*C. albicans*	14 mm at 100 mg/mL [197]

S4. Biological Activity Summary of Green Algae (Chlorophyta) (continued)				
U. lactuca	MeOH:DCM 1:1	Antioxidant	DPPH scavenging	IC_{50}: 16.5-18.7 μg/mL [198]
		Antibacterial	*B. cereus* (G+)	MIC 350-400 μg/mL [198]
		Antibacterial	*B. subtilis* (G+)	MIC 350-400 μg/mL [198]
		Antibacterial	*S. aureus* (G+)	MIC 350-400 μg/mL [198]
		Antibacterial	*M. luteus* (G+)	MIC 350-400 μg/mL [198]
		Antibacterial	*S. marcescencs* (G-)	MIC 350-400 μg/mL [198]
		Antibacterial	*K. pneumoniae* (G-)	MIC 350-400 μg/mL [198]
***U. lactuca* (cont.)**				
	Pure Compounds:			
	(C18 acid)	Antioxidant activators	ARE-Nrf2 antioxidant activator	EC_{50}: 6.0 μg/mL [199]
	(C16 acid)		ARE-Nrf2 antioxidant activator	EC_{50}: 17.7 μg/mL [199]
	3-O-β-D glucopyranosyl-clerosterol (**47**)	Antibacterial	*S. lactus* (G+)	25 mm at 200 μg/disk [38]
		Antibacterial	*B. subtilis* (G+)	15 mm at 200 μg/disk [38]
		Antibacterial	*P. putidu* (G-)	12 mm at 200 μg/disk [38]
		Antibacterial	*E. coli* (G-)	14 mm at 200 μg/disk [38]
		Antifungal	*F. oxysporium*	14 mm at 100 μg/disk [38]
		Antifungal	*Rhizoctonia solani*	7 mm at 100 μg/disk [38]
		Antifungal	*P. digitatum*	10 mm at 100 μg/disk [38]
		Antifungal	*S. cervisiae*	16 mm at 100 μg/disk [38]
		Antifungal	*K. lactis*	11 mm at 100 μg/disk [38]
		Anti-inflammatory	Mouse Ear Oedema	62.25% at 1000 μg/ear [38]

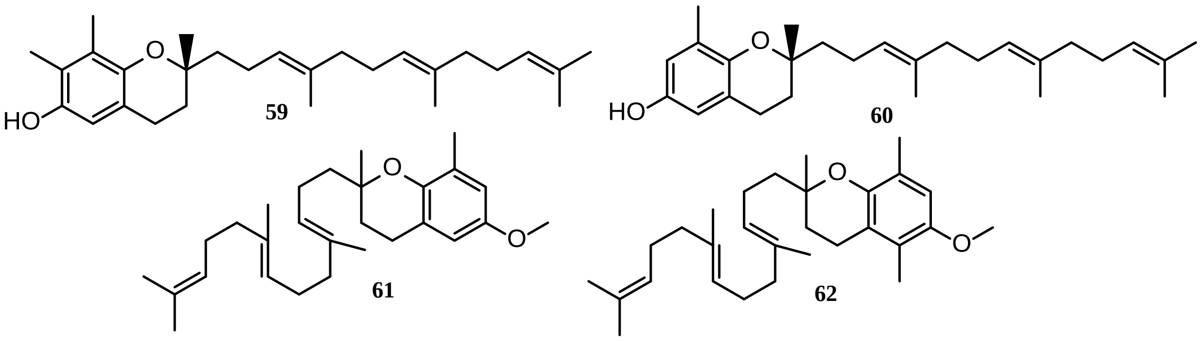

S8. Tocotrienols Isolated from Brown Algae.

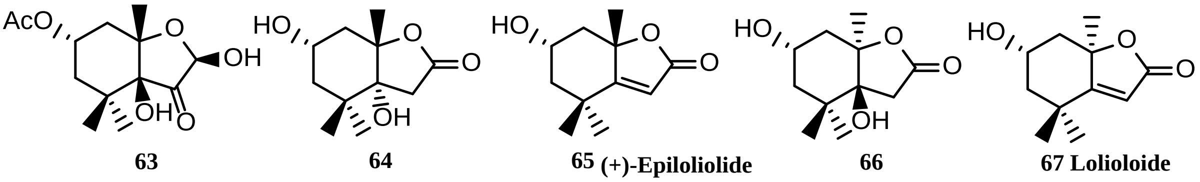

S9. Monoterpenes Isolated from Brown Algae.

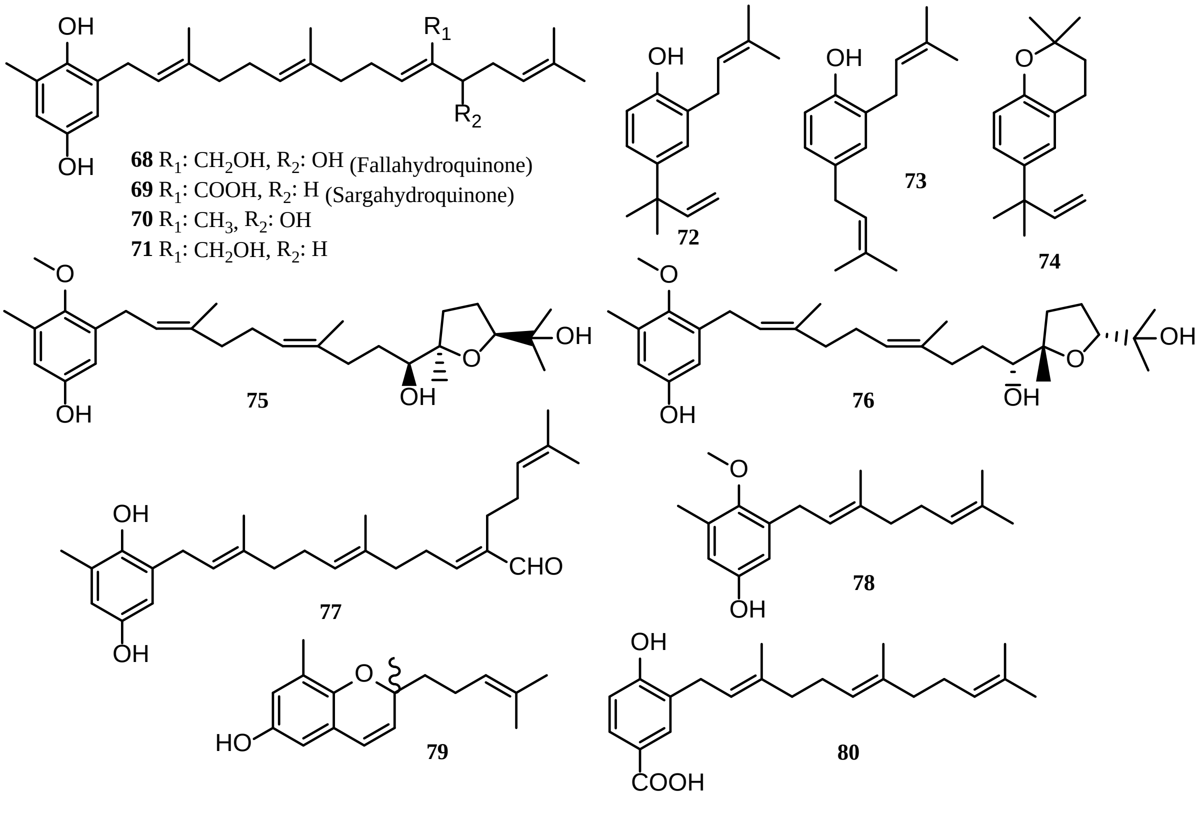

S10. Prenylated Phenols Isolated from Brown Algae.

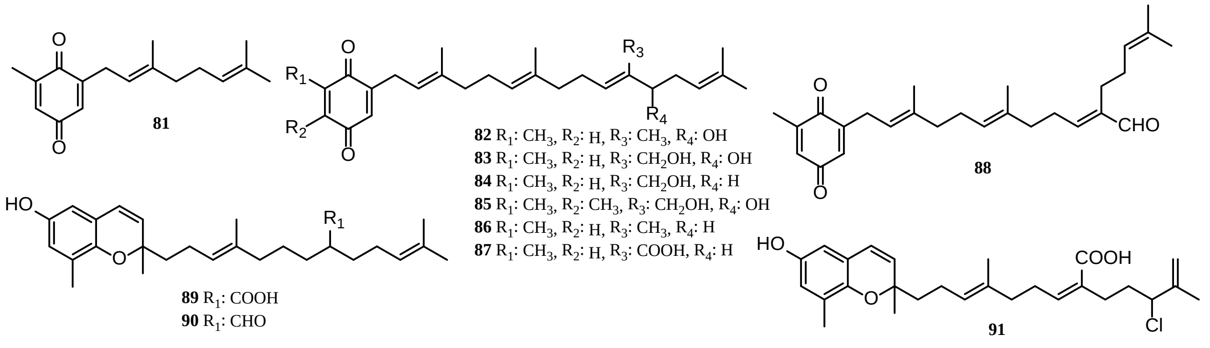

S11. Meroditerpenoids Isolated from Brown Algae.

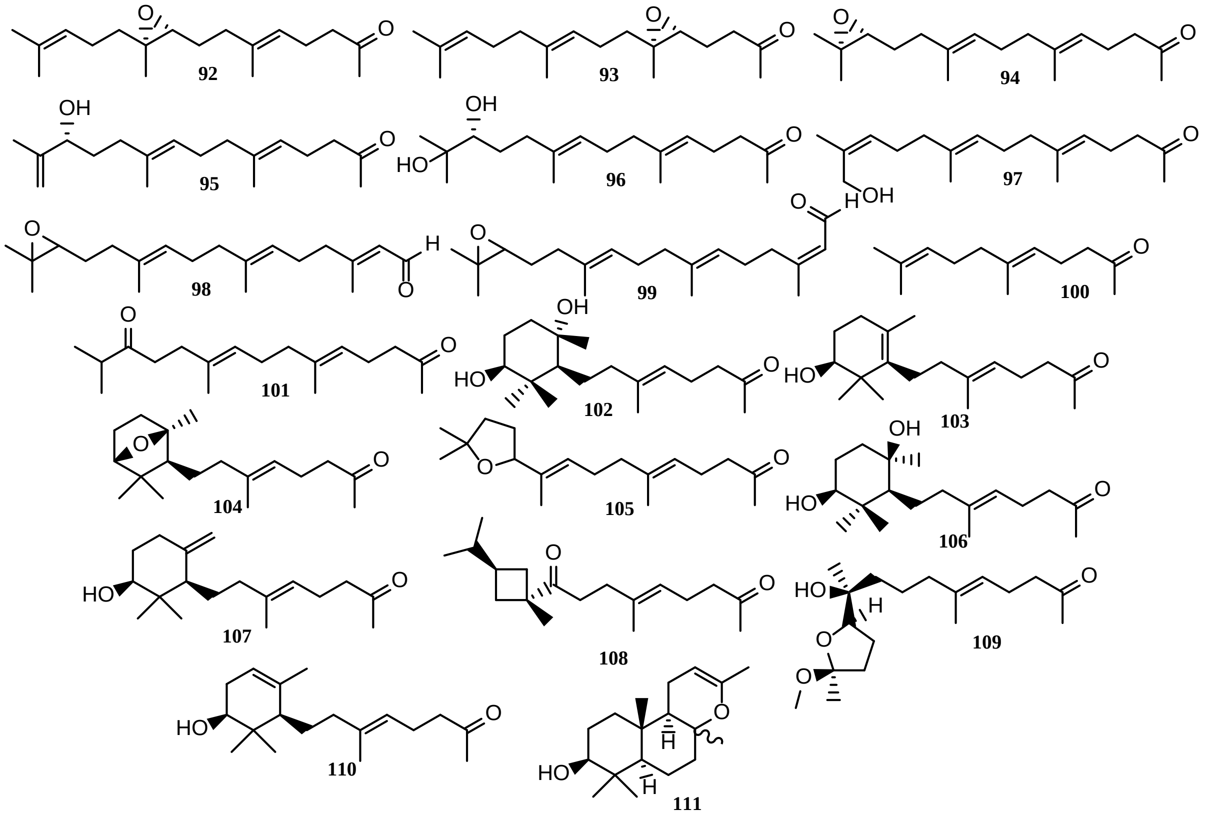

S12. Prenylated Acetones and Aldehydes Isolated from Brown Algae.

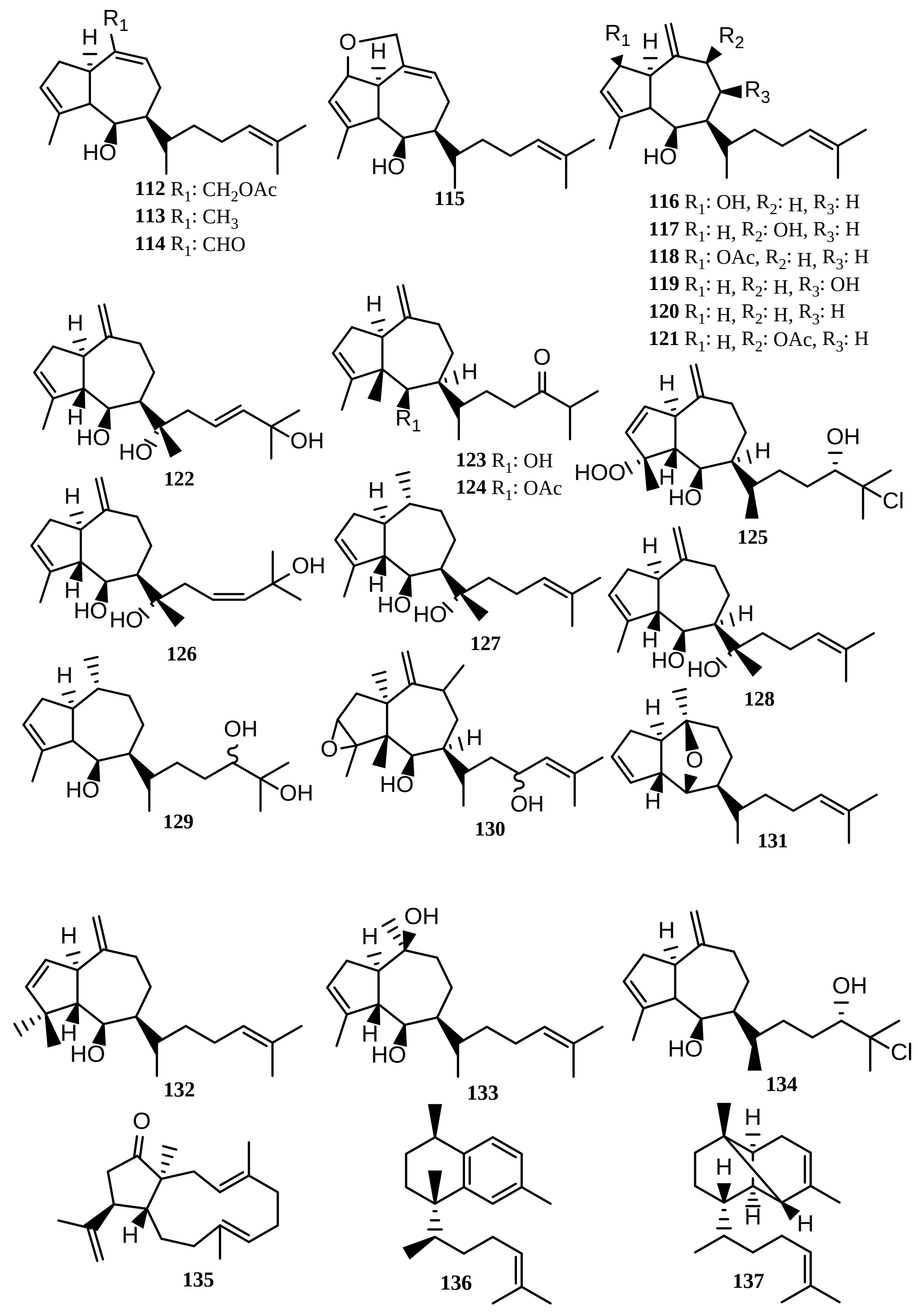

S13. Diterpenoids Isolated from Brown Algae.

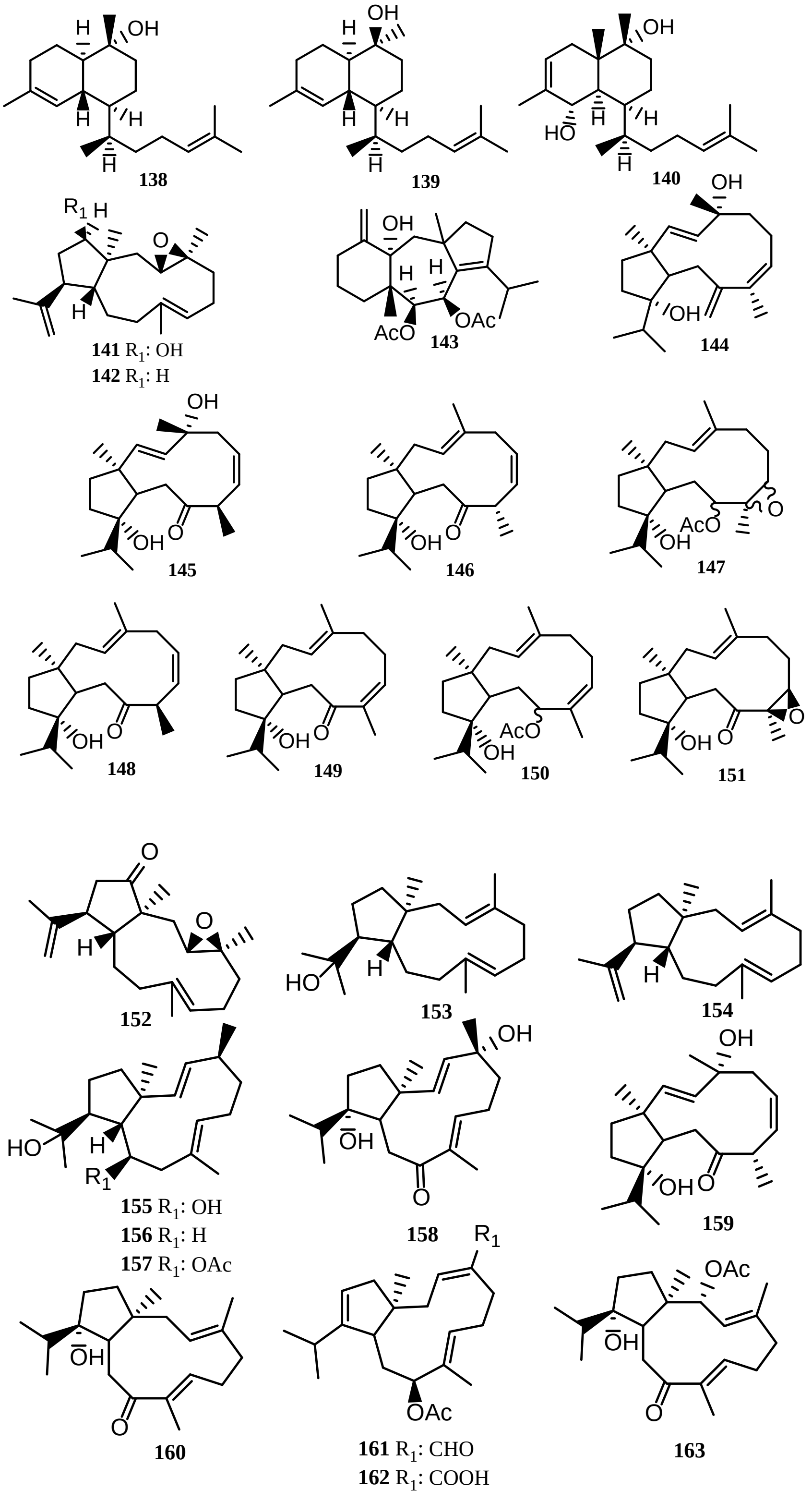

S14. Diterpenoids Isolated from Brown Algae (continued).

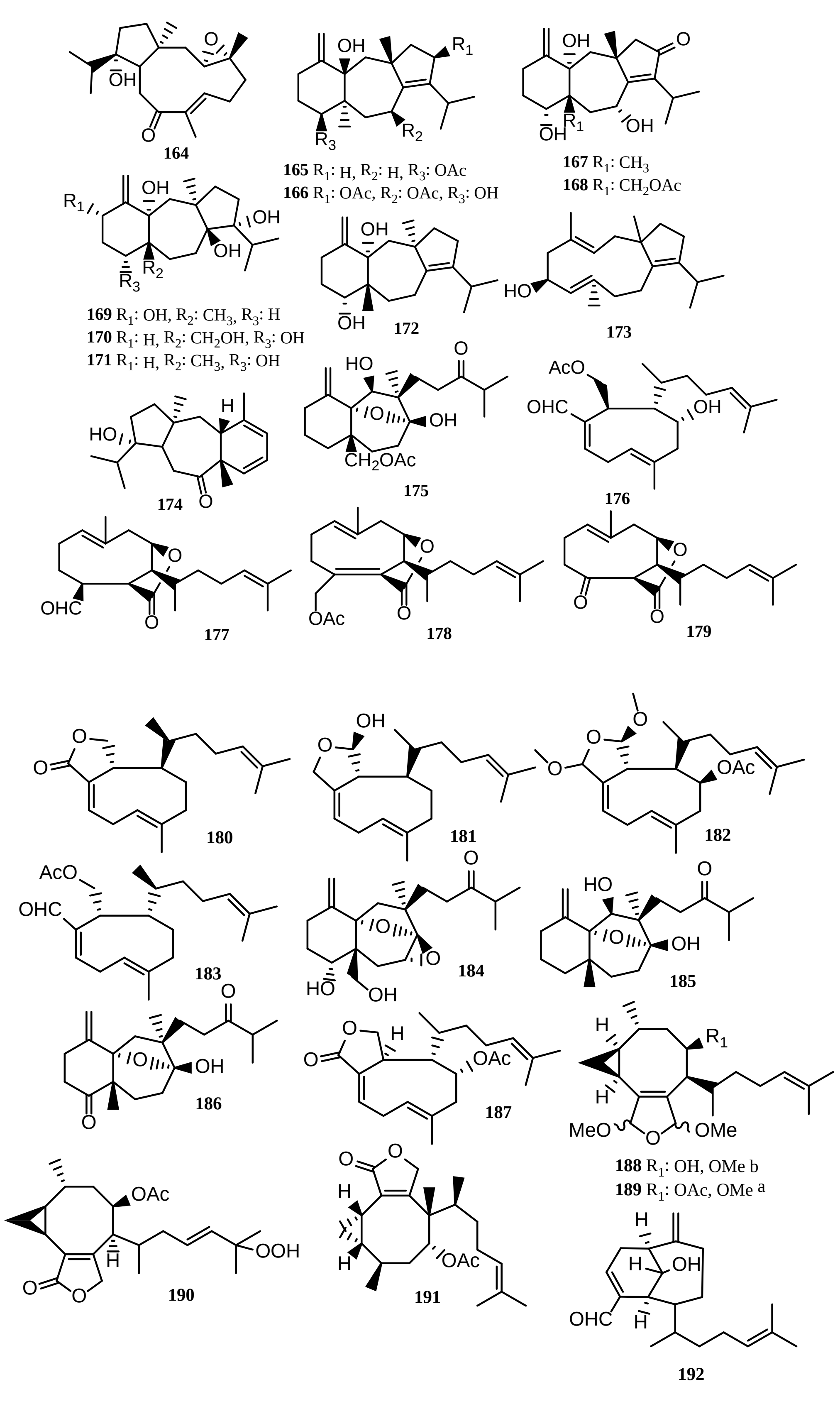

S15. Diterpenoids Isolated from Brown Algae (continued).

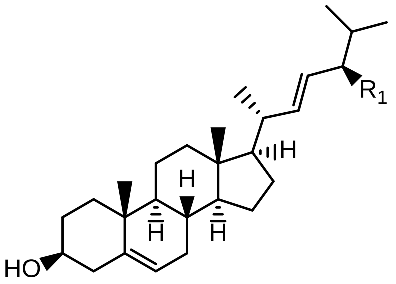

193 R_1: CH_3 (Brassicasterol)
194 R_1: H (Dehydrocholesterol)

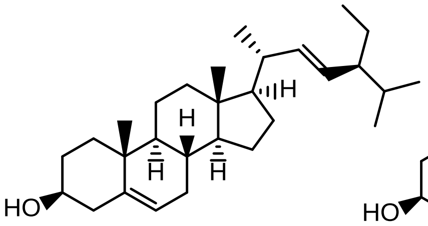

195 (Poriferasterol)

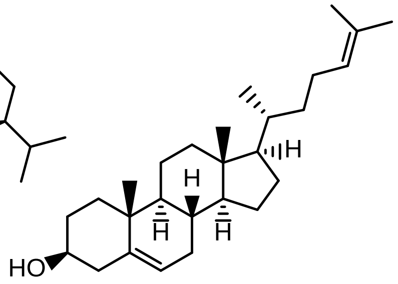

196 (Desmosterol)

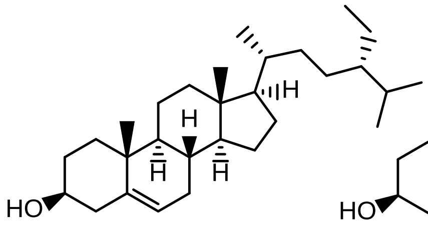

197

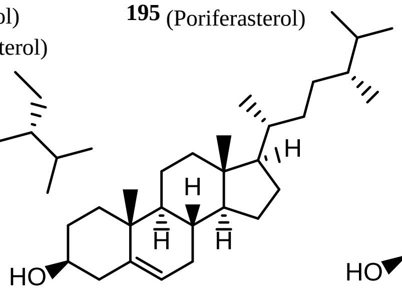

198 (Campesterol)

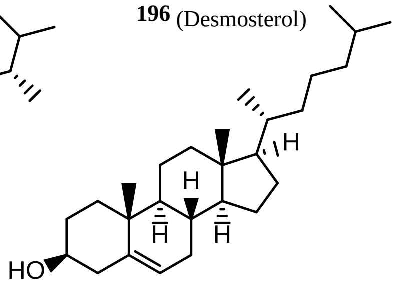

199 (Cholesterol)

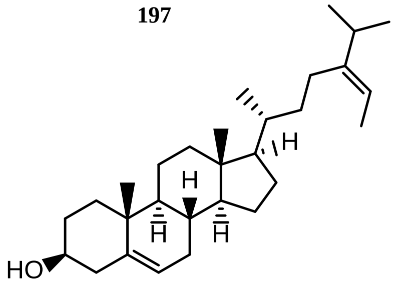

200 (Fucosterol)

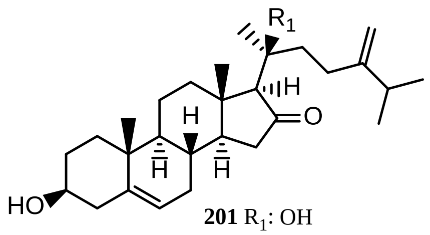

201 R_1: OH
202 R_1: H

S16. Steroids/sterols Isolated from Brown Algae.

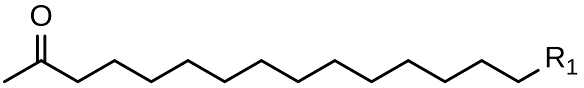

203 R_1: H
204 R_1: CH_2CH_3

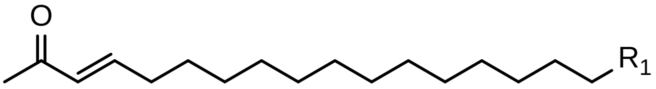

205 R_1: H
206 R_1: CH_3
207 R_1: CH_2CH_3
208 R_1: $(CH_2)_2CH_3$
209 R_1: $(CH_2)_3CH_3$

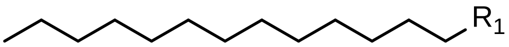

210 R_1: H
211 R_1: CH_3
212 R_1: CH_2CH_3
213 R_1: $(CH_2)_2CH_3$
214 R_1: $(CH_2)_3CH_3$

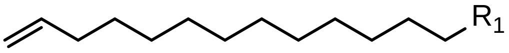

215 R_1: H
216 R_1: CH_3
217 R_1: CH_2CH_3
218 R_1: $(CH_2)_2CH_3$
219 R_1: $(CH_2)_3CH_3$

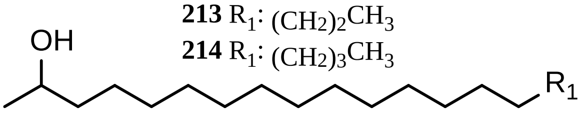

220 R_1: H
221 R_1: CH_2CH_3

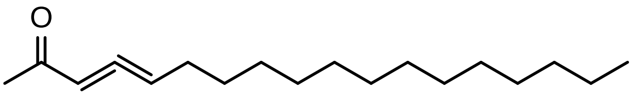

222

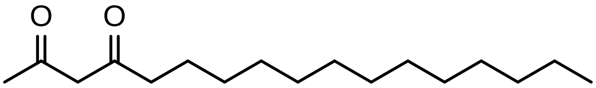

223

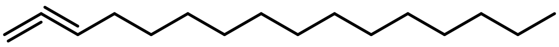

224

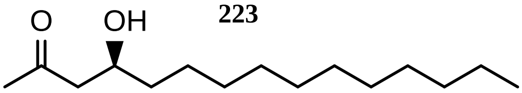

225

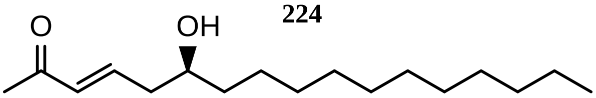

226

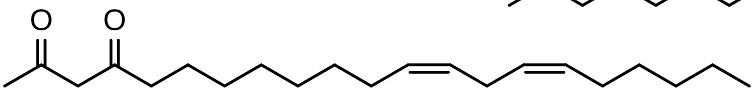

227

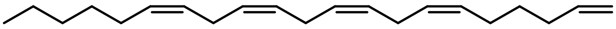

228

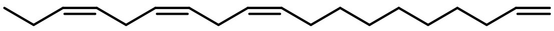

229

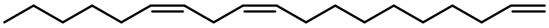

230

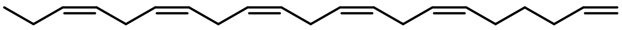

231

S17. Lipids and Polyenes Isolated from Brown Algae.

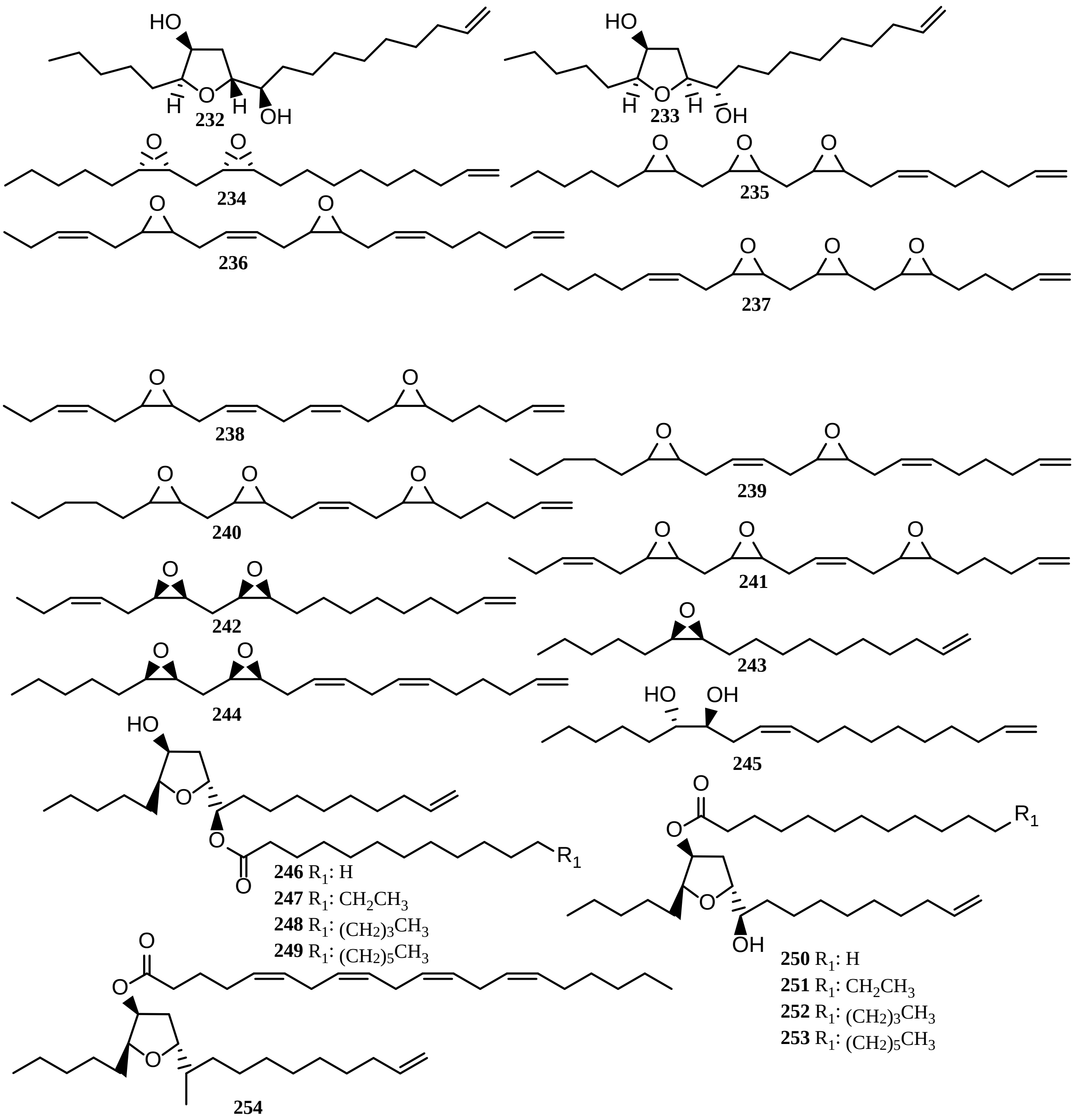

S18. Oxylipids and Epoxylipids Isolated from Brown Algae.

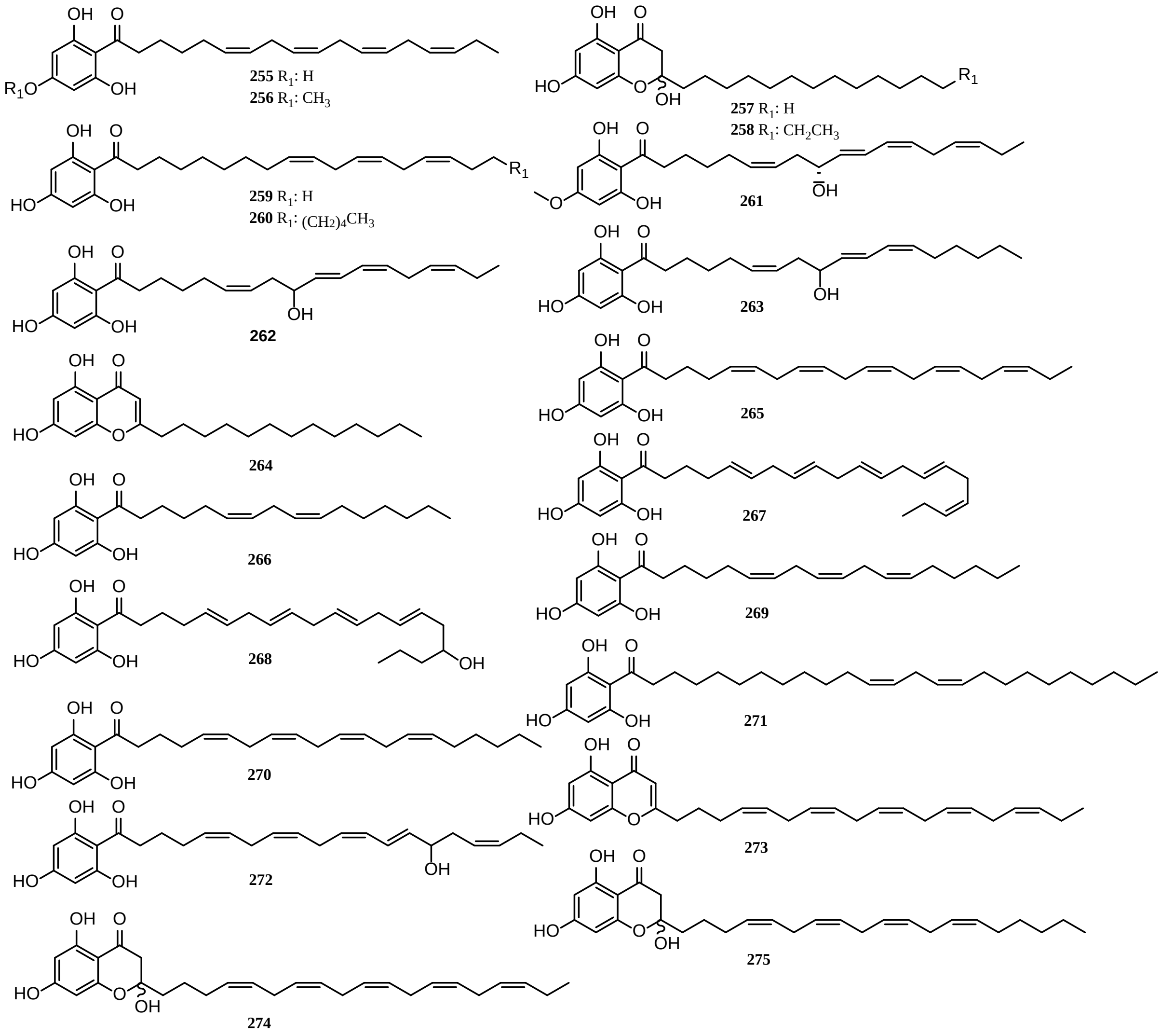

S19. Phloroglucinols Isolated from Brown Algae.

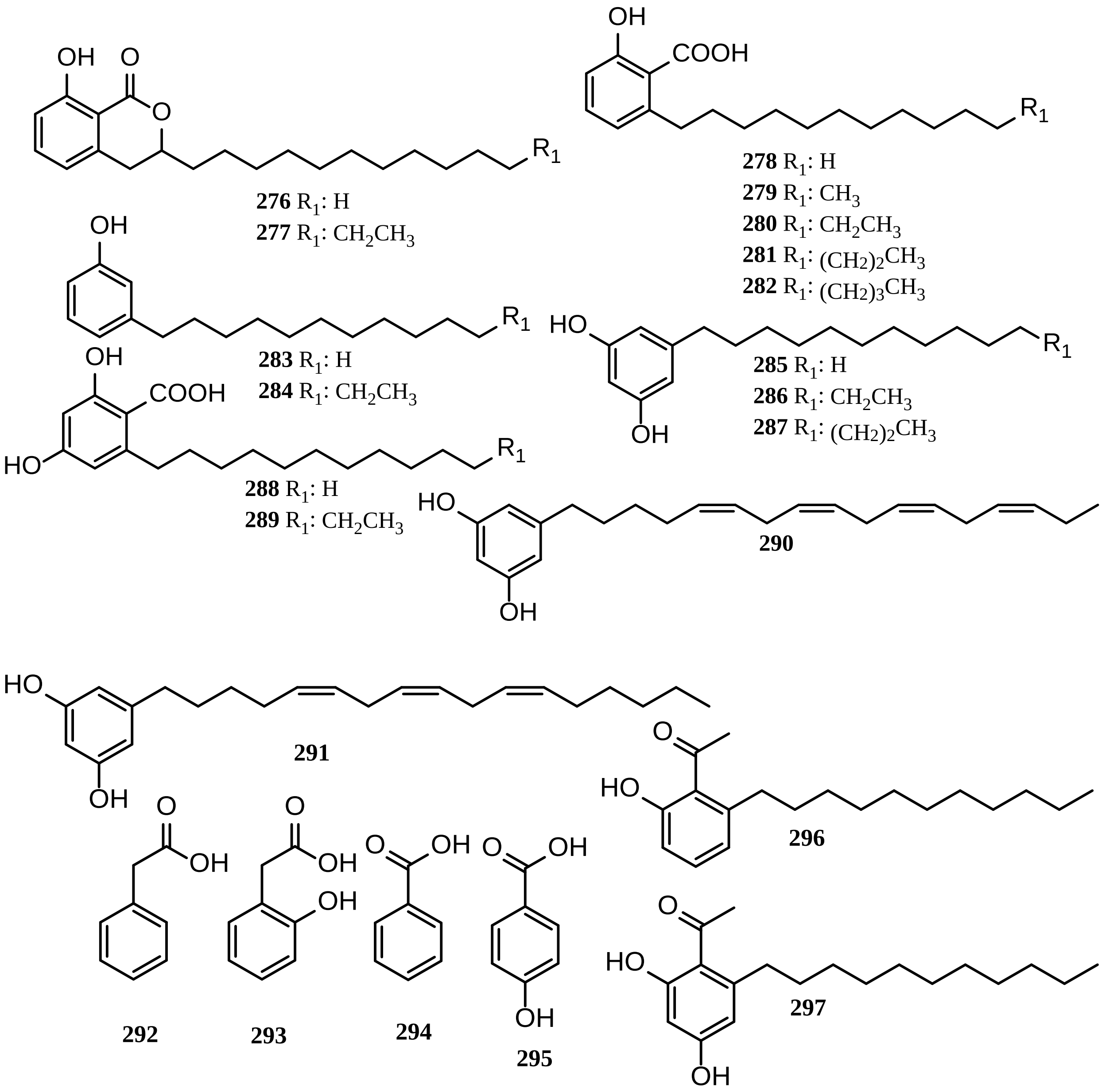

S20. Phenols/phenolic acids/resorcinols Isolated from Brown Algae.

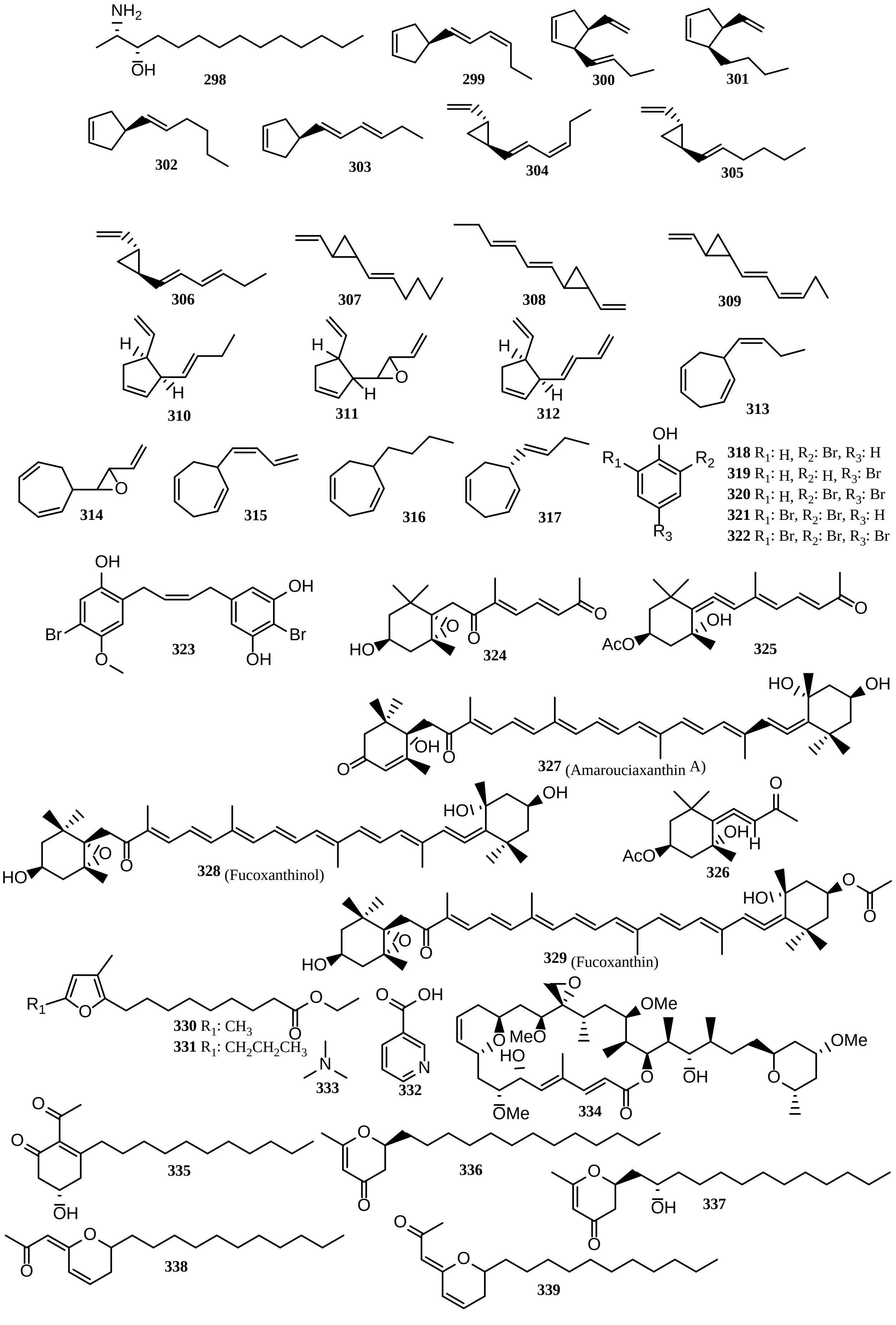

S21. Miscellaneous Compounds Isolated from Brown Algae.

S22. Biological Activity Summary of Brown Algae (Ochrophyta).

Species	Extract/Pure Compound	Activity	Assay	Potency [Ref]
C. spongiosum	Crude Extract:	Antioxidant	Hydroxyl Scavenging	EC_{25}: 0.54 mg/mL [175]
	Ethanol	Antioxidant	Nitric Oxide Scavenging	EC_{25}: 3.19 mg/mL [175]
C. sinuosa	Pure Compounds:			
		Antitumour	P388	IC_{50}: 10 µg/mL [105]
	Colpol (**323**)	Antitumour	A549	IC_{50}: 10 µg/mL [105]
		Antitumour	HT-29	IC_{50}: 10 µg/mL [105]
		Antitumour	CV-1	IC_{50}: 10 µg/mL [105]
	Crude Extracts:			
		Antibacterial	*S. aureus* (G+)	9 mm at 1.5 mg/disk [200]
	Methanol	Antibacterial	*MRSA* (G+)	8mm at 1.5 mg/disk [200]
		Antibacterial	*P. vulgaris* (G-)	6.5 mm at 1.5 mg/disk [200]
		Antibacterial	*S. aureus* (G+)	7.5 mm at 1.5 mg/disk [200]
	Dichloromethane	Antibacterial	*MRSA* (G+)	7.5 mm at 1.5 mg/disk [200]
		Antibacterial	*P. vulgaris* (G+)	8.5 mm at 1.5 mg/disk [200]
		Antibacterial	*B. subtillus* (G+)	6.5 mm at 1.5 mg/disk [200]
		Antibacterial	*P. vulgaris* (G-)	6.5 mm at 1.5 mg/disk [200]
	Hexane	Antibacterial	*S. aureus* (G+)	7 mm at 1.5 mg/disk [200]
		Antitumour	P388	IC_{50}: 20 µg/mL [105]
		Antitumour	A549	IC_{50}: 20 µg/mL [105]
	MeOH:DCM	Antitumour	HT-29	IC_{50}: 20 µg/mL [105]
	1:1	Antitumour	CV-1	IC_{50}: 20 µg/mL [105]
	PBS (Phosphate Buffered Saline)	Antitumour	L1210	0.1-0.3 (Ratio of viable Leukemia Cells in test compared to control) [201]
	Fucoxanthin Extract	Antitumour	Breast Cancer (MDA-MB-231)	varying [202]
C. moniliformis	Mixture:			
	(**107** + **110**)	Antitumour	P388	IC_{50}: 45 µM at 1 mg/mL [69]
	(**107** + **110**)	Antifungal	*T. mentagrophytes*	4 mm at 1 mg/mL [69]
	Crude Extract:			
	MeOH:DCM	Antitumour	P388	IC_{50}: 45 ng/mL [69]
	3:1			
C. monilifera	Pure Compounds:			
	(**263**)	Antibacterial	*S. pyogenes* (G+)	1 mm at 20 µg/disk [54]
	(**266**)	Antibacterial	*S. pyogenes* (G+)	1 mm at 20 µg/disk [54]
	δ-tocotrienol (**60**)	Anticancer	Pancreatic ductal cancer	significant [203]
		Anticancer	Lung cancer	Dose + time dependant [204]
	γ-tocotrienol (**59**)	Anticancer	Colon cancer (SW620 + HCT-8)	Dose dependant [205]
		Anticancer	3T3-L1 cells	Dose + time dependant [206]
	Crude Extracts:			
	Ethanol	Antioxidant	DPPH scavenging	~ (2018)[207]
	Dichloromethane	Antibacterial	*S. aureus* (G+)	1 mm at 20 µg/disk [54]
		Antibacterial	*MRSA* (G+)	2 mm at 20 µg/disk [54]
		Antibacterial	*S. pyogenes* (G+)	6 mm at 20 µg/disk [54]
C. platylobium	Pure Compounds:			
	δ-tocotrienol (**60**)	Anticancer	Pancreatic ductal cancer	significant [203]
		Anticancer	Lung cancer	Dose + time dependant [204]
C. retroflexa	Crude Extracts:			
		Antibacterial	*S. aureus* (G+)	2 mm at 20 µg/disk [89]
	Dichloromethane	Antibacterial	*MRSA* (G+)	3 mm at 20 µg/disk [89]
		Antibacterial	*P. aeruginosa* (G-)	4 mm at 20 µg/disk [89]
		Antibacterial	*S. aureus* (G+)	1 mm at 20 µg/disk [89]
	Methanol	Antibacterial	*MRSA* (G+)	4 mm at 20 µg/disk [89]
		Antibacterial	*P. aeruginosa* (G-)	6 mm at 20 µg/disk [89]
		Antibacterial	*E. coli* (G-)	1 mm at 20 µg/disk [89]
C. retorta	Pure Compounds:			
	δ-tocotrienol (**60**)	Anticancer	Pancreatic ductal cancer	significant [203]
		Anticancer	Lung cancer	Dose + time dependant [204]

S22. Biological Activity Summary of Brown Algae (Ochrophyta) (continued)

C. retorta	(**228**)	Lipoxygenase inhibition	Lipoxygenase	IC_{50}: 40 µM [81]
	(**231**)			IC_{50}: 5.0 µM [81]
C. siliquosa	Pure Compounds:			
	δ-tocotrienol (**60**)	Anticancer	Pancreatic ductal cancer	significant [203]
		Anticancer	Lung cancer	Dose + time dependant [204]
C. subfarcinata	Pure Compounds:			
	(**255**)	Antibacterial	*S. pyogenes* (G+)	1 mm at 20 µg/disk [54]
	(**256**)	Antibacterial	*S. aureus* (G+)	1 mm at 20 µg/disk [54]
		Antibacterial	*P. aeruginosa* (G-)	1 mm at 20 µg/disk [54]
	(**259**)	Antibacterial	*S. pyogenes* (G+)	1 mm at 20 µg/disk [54]
	δ-tocotrienol (**60**)	Anticancer	Pancreatic ductal cancer	significant [203]
		Anticancer	Lung cancer	Dose + time dependant [204]
	Crude Extracts:			
		Antibacterial	*P. aeruginosa* (G-)	1 mm at 20 µg/disk [89]
	MeOH:DCM	Antifungal	*C. albicans 14053*	3 mm at 20 µg/disk [89]
	3:1	Antifungal	*T. mentagrophytes*	3 mm at 20 µg/disk [54]
C. torulosa	Pure Compounds:			
	(**228**)	Lipoxygenase inhibition	Lipoxygenase	IC_{50}: 40 µM [81]
	(**231**)			IC_{50}: 5.0 µM [81]
		Antitumour	SMMC7721	IC_{50}: 31.45 µg/mL [96]
	(**286**)	Antitumour	K562	IC_{50}: 35.27 µg/mL [96]
		Antitumour	HeLa	IC_{50}: 24.28 µg/mL [96]
L. variegata	Pure Componds:			
		Antibacterial	*E. faecalis* (G+)	-10 % vs ciprofloxacin at 100 µg/mL [78]
	(**227**)	Antibacterial	*E. coli* (G-)	-2.4 % vs ciprofloxacin at 100 µg/mL [78]
		Antibacterial	*S. aureus* (G+)	-17 % vs ciprofloxacin at 100 µg/mL [78]
		Antibacterial	*E. faecalis* (G+)	-1 % vs ciprofloxacin at 100 µg/mL [78]
	(**296**)	Antibacterial	*E. coli* (G-)	-3.3 % vs ciprofloxacin at 100 µg/mL [78]
		Antibacterial	*S. aureus* (G+)	65 % vs ciprofloxacin at 100 µg/mL [78]
		Antibacterial	*E. faecalis* (G+)	-9.8 % vs ciprofloxacin at 100 µg/mL [78]
	(**297**)	Antibacterial	*E. coli* (G-)	-1.0 % vs ciprofloxacin at 100 µg/mL [78]
		Antibacterial	*S. aureus* (G+)	17 % vs ciprofloxacin at 100 µg/mL [78]
		Antifungal	*D. salina*	IC_{50}: 0.034 µg/mL
		Antifungal	*L. thalassiae*	IC_{50}: 0.135 µg/mL
	(**334**)	Antifungal	*C. albicans*	IC_{50}: 1.3 µg/mL
		Antifungal	*C. albicans* (amphotericin-resistant strain)	IC_{50}: 0.5 µg/mL
		Antitumour	Human colon tumour cell line HCT-116	IC_{50}: 0.03 µg/mL
		Antibacterial	*E. faecalis* (G+)	23 % vs ciprofloxacin at 100 µg/mL [78]
	(**335**)	Antibacterial	*E. coli* (G-)	29 % vs ciprofloxacin at 100 µg/mL [78]
		Antibacterial	*S. aureus* (G+)	100 % vs ciprofloxacin at 100 µg/mL [78]
		Antibacterial	*E. faecalis* (G+)	23 % vs ciprofloxacin at 100 µg/mL [78]
	(**337**)	Antibacterial	*E. coli* (G-)	27 % vs ciprofloxacin at 100 µg/mL [78]
		Antibacterial	*S. aureus* (G+)	-8.5 % vs ciprofloxacin at 100 µg/mL [78]
		Antibacterial	*E. faecalis* (G+)	21 % vs ciprofloxacin at 100 µg/mL [78]
	(**338**)	Antibacterial	*E. coli* (G-)	15 % vs ciprofloxacin at 100 µg/mL [78]
		Antibacterial	*S. aureus* (G+)	-95 % vs ciprofloxacin at 100 µg/mL [78]
		Antibacterial	*E. faecalis* (G+)	19 % vs ciprofloxacin at 100 µg/mL [78]
	(**339**)	Antibacterial	*E. coli* (G-)	25 % vs ciprofloxacin at 100 µg/mL [78]
		Antibacterial	*S. aureus* (G+)	-57 % vs ciprofloxacin at 100 µg/mL [78]

S22. Biological Activity Summary of Brown Algae (Ochrophyta) (continued)

N. anomala	Pure Compounds:			
	(**228**)	Lipoxygenase inhibition	Lipoxygenase	IC_{50}: 40 μM [81]
	(**231**)			IC_{50}: 5.0 μM [81]
	(**232**)	Nematocide	*T. colubriformis* and *H. contortus*	Significant [82]
	(**233**)	Nematocide		Significant [82]
P. caudata	Pure Compounds:			
	(**72**)	Antifungal	*Cr. neoformans* and *C. albicans*	MIC: 3.1 μg/mL [62]
		Antibacterial	*B. subtilus*	MIC: 6.2 μg/mL [62]
S. linearfolium	Pure Compound:			
	Fucosterol (**200**)	Antiplasmodial	*P. falciparum* (Malaria)	IC_{50}: 7.48 μg/mL [76]
S. vestitum	Crude Extract:			
		Antioxidant	ABTS	183.38 mg TE g^{-1} extract 1.0 mg/mL[73]
	Ethanol	Antioxidant	DPPH	209.50 mg TE g^{-1} extract 1.0 mg/mL [73]
		Antioxidant	FRAP	283.71 mg TE g^{-1} extract 1.0 mg/mL [73]
	MeOH:DCM	Antibacterial	*P. aeruginosa* (G-)	3 mm at 20 μg/disk [89]
	3:1	Antifungal	*C. albicans 14053*	3 mm at 20 μg/disk [89]
		Antifungal	*T. mentagrophytes*	2 mm at 20 μg/disk [89]
S. lomentaria	Crude Extract:			
		Antibacterial	*B. subtilis* (G+)	7.5 mm at 1.5 mg/disk [200]
	Methanol	Antibacterial	*S. aureus* (G+)	7.5 mm at 1.5 mg/disk [200]
		Antibacterial	*MRSA* (G+)	7 mm at 1.5 mg/disk [200]
		Antibacterial	*B. subtilis* (G+)	8.5 mm at 1.5 mg/disk [200]
		Antibacterial	*S. aureus* (G+)	8.5 mm at 1.5 mg/disk [200]
		Antibacterial	*MRSA* (G+)	7.5 mm at 1.5 mg/disk [200]
	Dichloromethane	Antibacterial	*E. aerogenes* (G-)	6.5 mm at 1.5 mg/disk [200]
		Antibacterial	*E. coli* (G-)	9 mm at 1.5 mg/disk [200]
		Antibacterial	*E. coli* hemorrhagic (G-)	9.5 mm at 1.5 mg/disk [200]
		Antibacterial	*P. vulgaris* (G-)	7.5 mm at 1.5 mg/disk [200]
		Antibacterial	*S. typhimurium* (G-)	6.5 mm at 1.5 mg/disk [200]
		Antifungal	*C. albicans*	6.5 mm at 1.5 mg/disk [200]
		Antibacterial	*B. subtilis* (G+)	7.5 mm at 1.5 mg/disk [200]
		Antibacterial	*S. aureus* (G+)	8 mm at 1.5 mg/disk [200]
		Antibacterial	*MRSA* (G+)	11 mm at 1.5 mg/disk [200]
	Hexane	Antibacterial	*E. aerogenes* (G-)	7 mm at 1.5 mg/disk [200]
		Antibacterial	*E. coli* (G-)	7 mm at 1.5 mg/disk [200]
		Antibacterial	*E. coli* hemorrhagic (G-)	7 mm at 1.5 mg/disk [200]
		Antibacterial	*P. vulgaris* (G-)	8 mm at 1.5 mg/disk [200]
		Antibacterial	*S. typhimurium* (G-)	8 mm at 1.5 mg/disk [200]
		Antifungal	*C. albicans*	7.5 mm at 1.5 mg/disk [200]
	PBS	Antitumour	L1210 lymphoid leukemia	0.03-0.1(Ratio of viable Leukemia Cells in test compared to control) [201]
	Aqueous	Antitumour	Ehrlich carcinoma	69.8% inhibition *in vivo* [208]
S. fallax	Pure Compounds:			
	Sargaquinoic acid (**87**)	Antitumour	P388	IC_{50}: 17 μM at 1 mg/mL [60]
	Sargahydroquinoic acid(**69**)	Antitumour	P388	IC_{50}: 14 μM at 1 mg/mL [60]
	Sargaquinone (**86**)	Antitumour	P388	IC_{50}: 32 μM at 1 mg/mL [60]
	Fallahydroquinone (**68**)	Antitumour	P388	IC_{50}: >27-29 μM at 1 mg/mL [60]
	Fallaquinone (**83**)	Antitumour	P388	IC_{50}: >27-29 μM at 1 mg/mL [60]
	Fallachromenoic acid (**91**)	Antitumour	P388	IC_{50}: >27-29 μM at 1 mg/mL [60]
	Sargachromenol (**89**)	Antitumour	P388	IC_{50}: >27-29 μM at 1 mg/mL [60]
	Sargaquinoic acid (**87**)	Antibacterial	*B. subtilis* (G+)	Slight inhibition [60]
	Sargahydroquinoic acid (**69**)	Antibacterial	*B. subtilis* (G+)	Slight Inhibition [60]
	Crude Extracts:			
	MeOH:DCM	Antitumour	P388	IC_{50}: 6984 ng/mL at 50 mg/mL [60]
	3:1	Antiviral	*H. simplex,* Polio	Moderate [60]
		Antibacterial	*B. subtilis* (G+)	Moderate [60]

S22. Biological Activity Summary of Brown Algae (Ochrophyta) (continued)

S. paradoxum	Pure Compounds:			
	Fallahydroquinone (**68**)	Antibacterial	*S. pyogenes* (G+)	1 mm at 20 µg/disk [59]
		Antibacterial	*S. aureus* (G+)	1 mm at 20 µg/disk [59]
	Sargahydroquinoic acid(**69**)	Antibacterial	MRSA (G+)	1 mm at 20 µg/disk [59]
		Antibacterial	*P. aeruginosa* (G-)	2 mm at 20 µg/disk [59]
		Antibacterial	*S. pyogenes* (G+)	3 mm at 20 µg/disk [59]
	Paradoxyhydroquinone (**70**)	Antibacterial	*S. pyogenes* (G+)	3 mm at 20 µg/disk [59]
	(**71**)	Antibacterial	*S. pyogenes* (G+)	3 mm at 20 µg/disk [59]
	Fallaquinone (**83**)	Antibacterial	*S. pyogenes* (G+)	4 mm at 20 µg/disk [59]
	Sargaquinal (**88**)	Antibacterial	*P. aeruginosa* (G-)	3 mm at 20 µg/disk [59]
		Antibacterial	*S. pyogenes* (G+)	1 mm at 20 µg/disk [59]
	Paradoxquinol (**85**)	Antibacterial	*S. pyogenes* (G+)	1 mm at 20 µg/disk [59]
		Antibacterial	*S. aureus* (G+)	1 mm at 20 µg/disk [59]
	Sargaquinoic acid (**87**)	Antibacterial	MRSA (G+)	1 mm at 20 µg/disk [59]
		Antibacterial	*P. aeruginosa* (G-)	1 mm at 20 µg/disk [59]
		Antibacterial	*S. pyogenes* (G+)	3 mm at 20 µg/disk [59]
	Paradoxquinone (**82**)	Antibacterial	*S. pyogenes* (G+)	2 mm at 20 µg/disk [59]
	(**84**)	Antibacterial	*S. pyogenes* (G+)	2 mm at 20 µg/disk [59]
	Sargaquinone (**86**)	Antibacterial	*P. aeruginosa* (G-)	5 mm at 20 µg/disk [59]
	Crude Extracts:			
		Antibacterial	*S. aureus* (G+)	2 mm at 20 µg/disk [59]
	Dichloromethane	Antibacterial	MRSA (G+)	1 mm at 20 µg/disk [59]
		Antibacterial	*S. pyogenes* (G+)	5 mm at 20 µg/disk [59]
		Antibacterial	*S. aureus* (G+)	1 mm at 20 µg/disk [59]
		Antibacterial	MRSA (G+)	1 mm at 20 µg/disk [59]
	Methanol	Antibacterial	*P. aeruginosa* (G-)	10 mm at 20 µg/disk [59]
		Antibacterial	*S. pyogenes* (G+)	3 mm at 20 µg/disk [59]
		Antifungal	*C. albicans*	4 mm at 20 µg/disk [59]

S22. Biological Activity Summary of Brown Algae (Ochrophyta) (continued)

U. pinnatifida	Pure Compounds:			
	Fucosterol (**200**)	Antialzheimers	BACE1 inhibitor	IC$_{50}$: 64.12 µM [209]
	Fucoxanthin (**329**)	Antialzheimers	BACE1 inhibitor	IC$_{50}$: 5.31 µM [209]
	Galactofucan sulphate	Antiviral	*H. simplex* (HSV-1)	EC$_{50}$: 32µg/ mL [210]
		Antiviral	*H. simplex* (HSV-2)	EC$_{50}$: 32µg/ mL [210]
		Antiinflammatory	Edema	IC$_{50}$: 160 µg/ear [211]
	Stearidonic acid	Antiinflammatory	Erythema	IC$_{50}$: 314 µg/ear [211]
		Antiinflammatory	Blood Flow	IC$_{50}$: 235 µg/ear [211]
		Antiinflammatory	Edema	IC$_{50}$: 230 µg/ear [211]
	Eicosapentaenoic Acid	Antiinflammatory	Erythema	IC$_{50}$: 462 µg/ear [211]
		Antiinflammatory	Blood Flow	IC$_{50}$: 236 µg/ear [211]
		Antioxidant	DPPH scavenging	IC$_{50}$: 17 mM [57]
	Epiloliolide (**65**)	Antifungal	*Candida albicans*	6.5 mm at 800 µg/mL [57]
		Antibacterial	*E. coli* (G-)	7.0 mm at 800 µg/mL [57]
		Antibacterial	*MRSA* (G+)	8.2 mm at 800 µg/mL [57]
		Antibacterial	*S. aureus* (G+)	7.5 mm at 800 µg/mL [57]
	Loliolide (**67**)	Allelopathic	*E. crus-galli* root growth	IC$_{50}$: 16.46 µM [58]
	Crude Extracts:			
				IC$_{50}$: 0.92 mg/mL [212]
	80% Methanol	Antidiabetes	Alpha amylase inhibitor	IC$_{50}$: 0.81 mg/mL [212]
	70% Acetone	Antidiabetes	Alpha amylase inhibitor	IC$_{50}$: 2.07 mg/mL [212]
	Aqueous	Antidiabetes	Alpha amylase inhibitor	
				51.1 % [213]
	Methanol (sporophyll)	Antioxidant	DPPH Scavenging	8.3 % [213]
	Acetone (sporophyll)	Antioxidant	DPPH Scavenging	5.4 % [213]
	Chloroform (sporophyll)	Antioxidant	DPPH Scavenging	23.5 % [213]
	Ethyl acetate (sporophyll)	Antioxidant	DPPH Scavenging	
	PBS			0.03-0.1(Ratio of viable Leukemia Cells
		Antitumour	Lymphoid leukemia cells	in test compared to control) [201]
	Fucoidan extract			53.4% inhibition *in vivo* [208]
		Antitumour	Ehrlich carcinoma	
X. chondrophylla	Pure Compounds:			
		Antitumour	HL60	IC$_{50}$: 8.8 µM [99]
	Xestoaminol (**298**)	Antitumour	HEK	IC$_{50}$: 18 µM [99]
		Antibacterial	*S. aureus* (G+)	IC$_{50}$: 17 µM [99]
		Antifungal	*S. cerevisiae*	IC$_{50}$: 17.1 µM [99]
		Antitubercular	*M. tuberculosis* (H37Ra)	IC$_{50}$: 19.4 µM [99]
Z. angustata	Crude Extracts:			
	Ethanol	Antioxidant	DPPH Scavenging	9.4-9.6 µg GAE/10 µL [193]
Z. spiralis	Pure Compounds:			
	(**257**)	Antibacterial	*B. subtilis* (G+)	IC$_{50}$: 2.5-10 µM [91]
	(**258**)	Antibacterial	*B. subtilis* (G+)	IC$_{50}$: 2.5-10 µM [91]
	(**273**)	Antibacterial	*B. subtilis* (G+)	IC$_{50}$: 2.5-10 µM [91]
	(**275**)	Antibacterial	*B. subtilis* (G+)	IC$_{50}$: 2.5-10 µM [91]

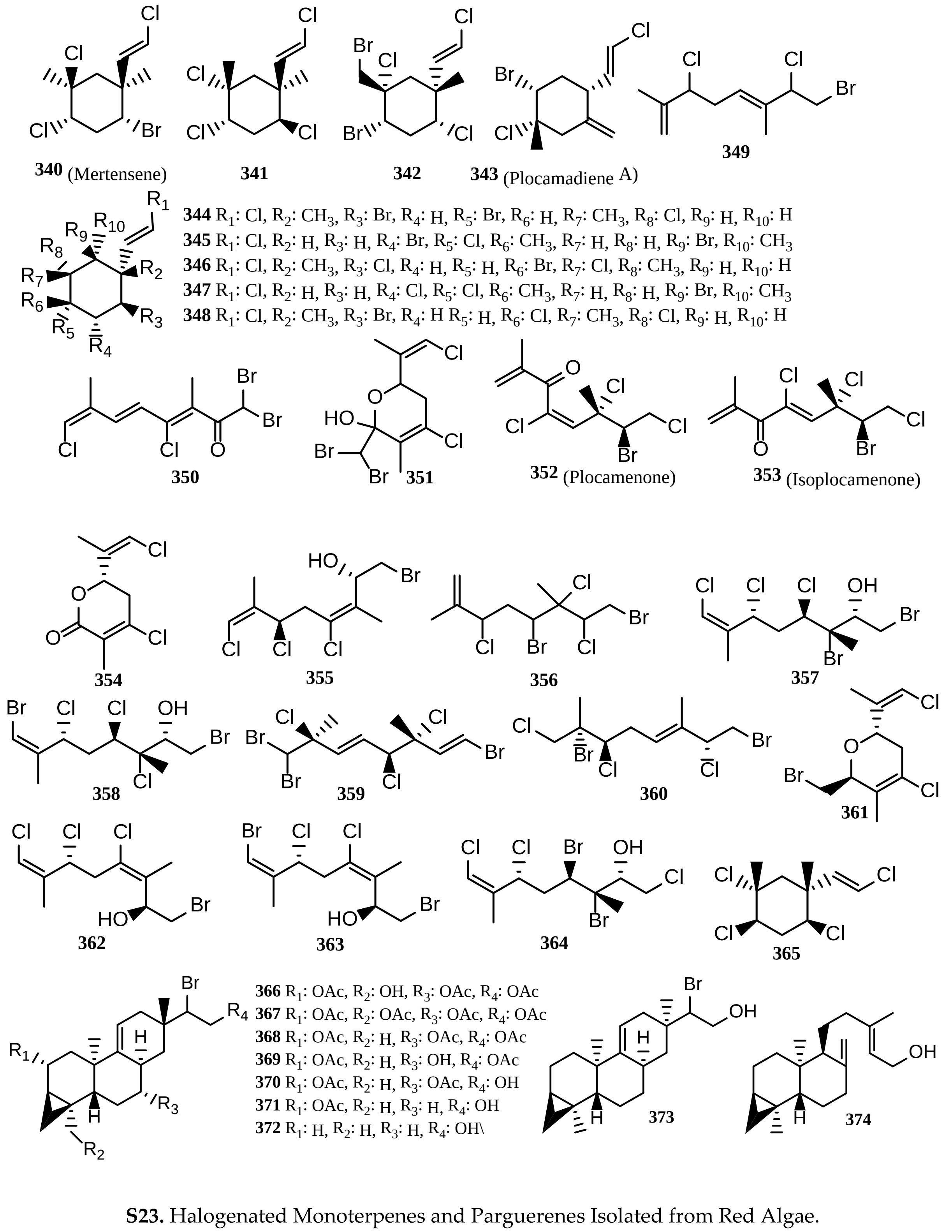

S23. Halogenated Monoterpenes and Parguerenes Isolated from Red Algae.

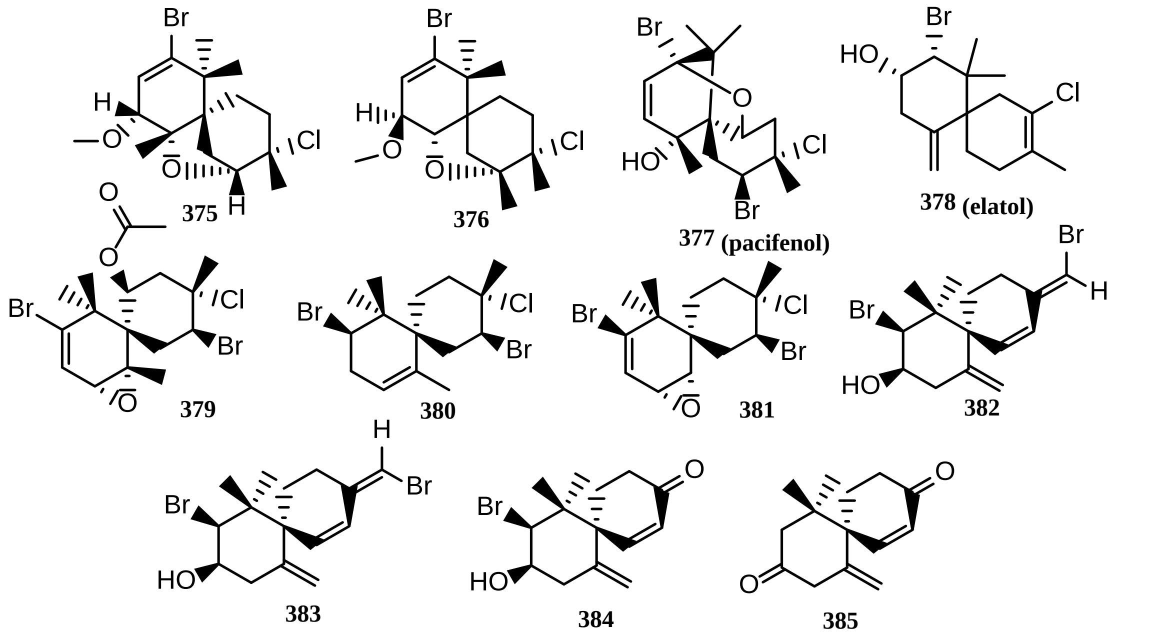

S24. Chamigrenes Isolated from Red Algae.

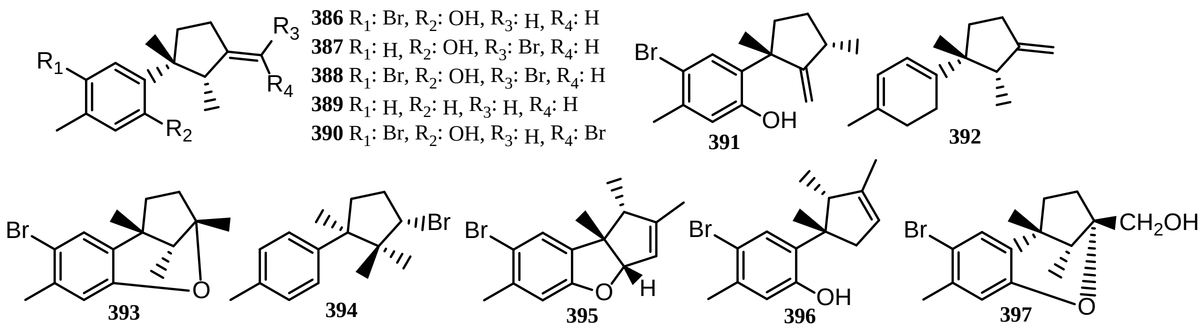

S25. Laurenes Isolated from Red Algae.

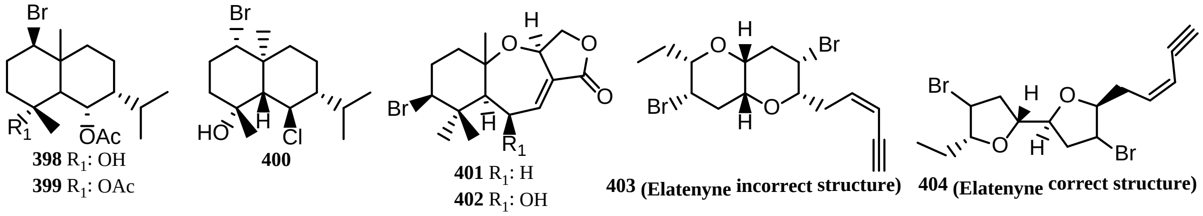

S26. Sesquiterpenes Isolated from Red Algae

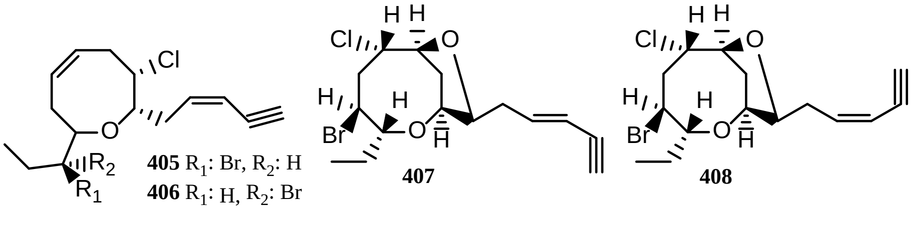

S27. Lauroxocanes Isolated from Red Algae

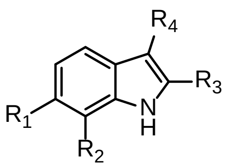

409 R_1: Br, R_2: Br, R_3: Br, R_4: Br
410 R_1: I, R_2: Br, R_3: Cl, R_4: Cl
411 R_1: I, R_2: Br, R_3: Br, R_4: Cl
412 R_1: I, R_2: Br, R_3: Cl, R_4: Br
413 R_1: Br, R_2: Br, R_3: Br, R_4: Cl
414 R_1: Br, R_2: Br, R_3: Cl, R_4: Br
415 R_1: Br, R_2: Br, R_3: Cl, R_4: Cl
416 R_1: I, R_2: Cl, R_3: Cl, R_4: Cl

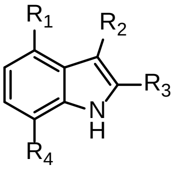

417 R_1: Cl, R_2: Cl, R_3: Br, R_4: I
418 R_1: Cl, R_2: Cl, R_3: Cl, R_4: Br
419 R_1: Cl, R_2: Cl, R_3: Cl, R_4: I
420 R_1: Br, R_2: Br, R_3: Br, R_4: Br
421 R_1: Br, R_2: Cl, R_3: Cl, R_4: Br

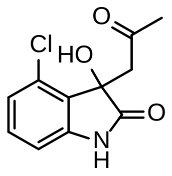

422

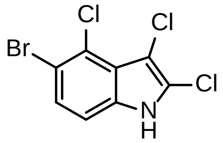

423

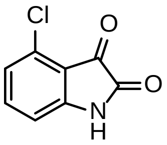

424

S28. Polyhalogenated Indoles Isolated from Red Algae.

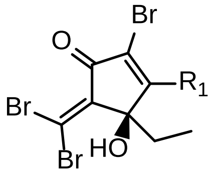

425 R_1: Br
426 R_1: OCH_3

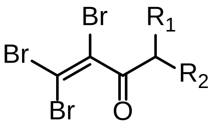

427 R_1: H, R_2: $(CH_2)_4CH_3$
428 R_1: Br, R_2: $(CH_2)_3CH_3$

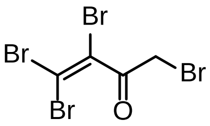

429

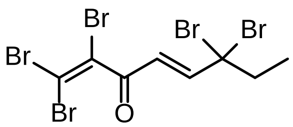

430

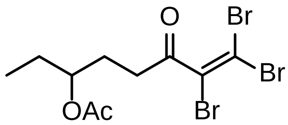

431

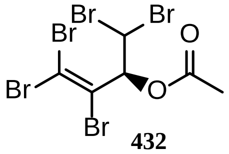

432

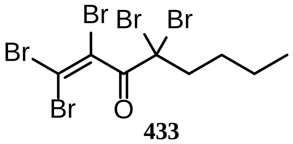

433

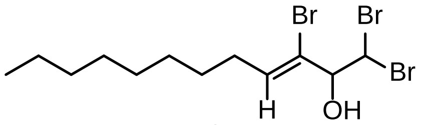

434

S29. Polyhalogenated Hydrocarbons Isolated from Red Algae.

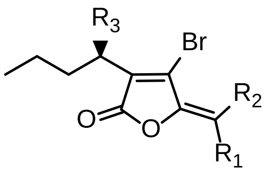

435 R$_1$: Br, **R$_2$:** H, **R$_3$:** OAc
436 R$_1$: H, **R$_2$:** Br, **R$_3$:** OH
437 R$_1$: Br, **R$_2$:** H, **R$_3$:** H
438 R$_1$: Br, **R$_2$:** Br, **R$_3$:** H
439 R$_1$: H, **R$_2$:** I, **R$_3$:** OH
440 R$_1$: I, **R$_2$:** H, **R$_3$:** OH
441 R$_1$: Br, **R$_2$:** H, **R$_3$:** OH
442 R$_1$: Br, **R$_2$:** H, **R$_3$:** OAc
443 R$_1$: H, **R$_2$:** Br, **R$_3$:** OAc
444 R$_1$: I, **R$_2$:** H, **R$_3$:** OAc
445 R$_1$: Br, **R$_2$:** Br, **R$_3$:** OH
446 R$_1$: Br, **R$_2$:** Br, **R$_3$:** OAc
447 R$_1$: Cl, **R$_2$:** H, **R$_3$:** OH
448 R$_1$: Br, **R$_2$:** Br, **R$_3$:** H
449 R$_1$: Br, **R$_2$:** H, **R$_3$:** H
450 R$_1$: H, **R$_2$:** Br, **R$_3$:** H
451 R$_1$: H, **R$_2$:** Cl, **R$_3$:** OH

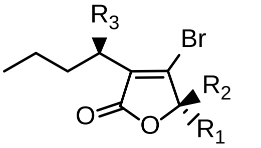

452 R$_1$: OCH$_3$, **R$_2$:** CHBr$_2$, **R$_3$:** OAc
453 R$_1$: OCH$_3$, **R$_2$:** CH$_2$Br, **R$_3$:** OAc
454 R$_1$: CH$_2$Br, **R$_2$:** OCH$_3$, **R$_3$:** OAc
455 R$_1$: CH$_2$I, **R$_2$:** OCH$_3$, **R$_3$:** OAc
456 R$_1$: OCH$_3$, **R$_2$:** CH$_2$I, **R$_3$:** OH
457 R$_1$: CH$_2$Br, **R$_2$:** OCH$_3$, **R$_3$:** OH
458 R$_1$: CH$_2$I, **R$_2$:** OCH$_3$, **R$_3$:** OH
459 R$_1$: CH$_3$, **R$_2$:** OCH$_3$, **R$_3$:** OAc
460 R$_1$: CHBr$_2$, **R$_2$:** OCH$_3$, **R$_3$:** OAc
461 R$_1$: OCH$_3$, **R$_2$:** CH$_3$, **R$_3$:** OAc
462 R$_1$: CHBr$_2$, **R$_2$:** OCH$_3$, **R$_3$:** H

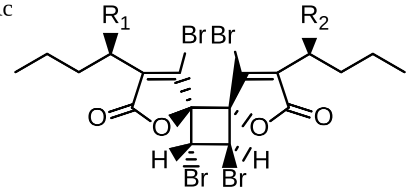

463 R$_1$: OAc, **R$_2$:** H
464 R$_1$: OAc, **R$_2$:** OAc
465 R$_1$: H, **R$_2$:** H

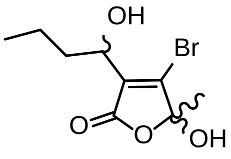

466

S30. Halogenated Furanones Isolated from Red Algae.

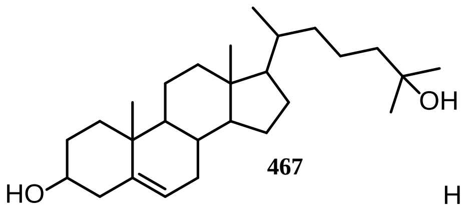

467

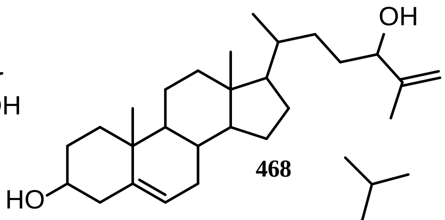

468

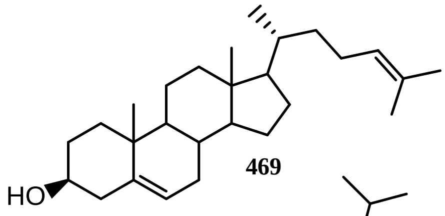

469

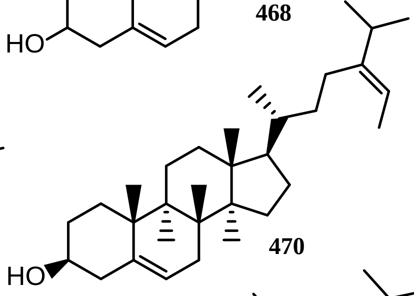

470

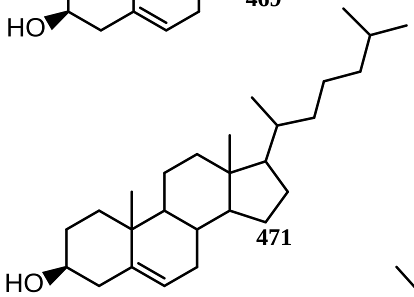

471

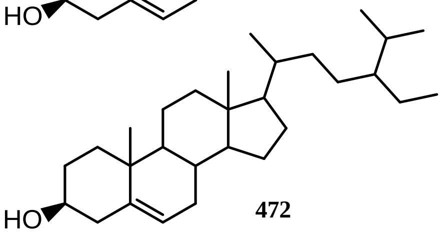

472

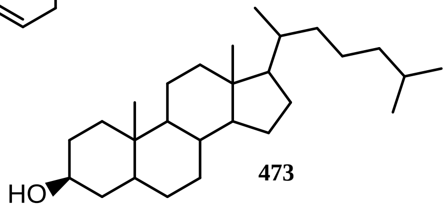

473

S31. Steroids Isolated from Red Algae.

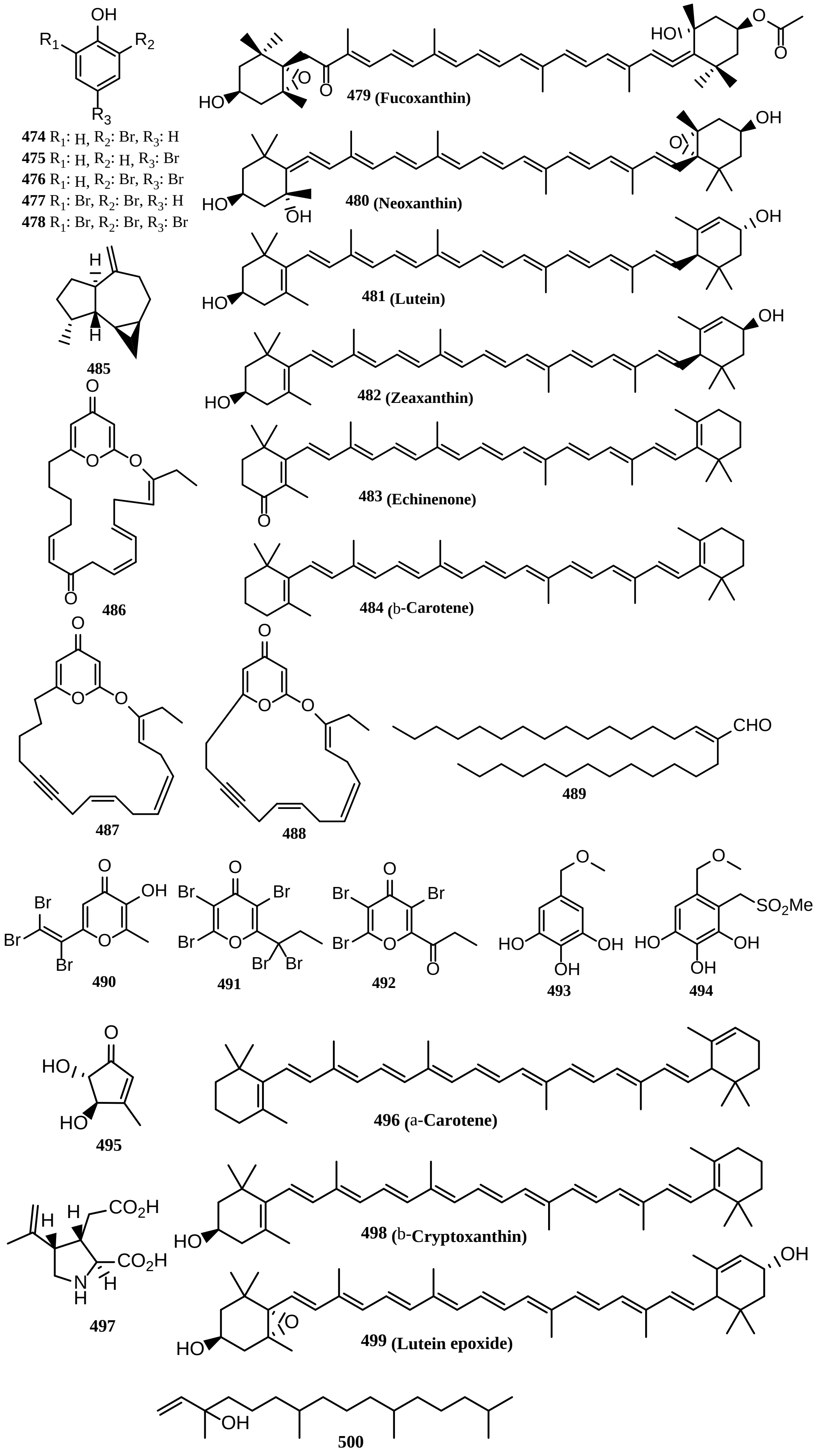

S32. Miscellaneous Classes of Compounds Isolated from Red Algae.

S33. Biological Activity Summary of Red Algae (Rhodophyta).

Species	Extract/Pure Compound	Activity	Assay	Potency [Ref]
C. officinales	Sulphated polysaccharide fraction	Antioxidant	Hydroxyl scavenging	IC_{50}: 1.44 mg/mL [214]
		Antioxidant	Superoxide scavenging	IC_{50}: 0.59 mg/mL [214]
		Antioxidant	DPPH scavenging	IC_{50}: 1.2 mg/mL [214]
D. pulchra	Pure Compounds:			
	427	Antimicrobial	*M. luteus* (G+)	Active at 5 µg [169]
		Antifungal	*P. oxalis*	5 mm at 5 µg [169]
		Antimicrobial	*B. subtilis* (G+)	Active at 5 µg [169]
	431	Antimicrobial	*E. coli* (G-)	Active at 5 µg [169]
		Antifungal	*P. oxalis*	9 mm at 5 µg [169]
		Anticancer	Tyrosine kinase inhibitor	%REA: 31.7% at 200 µg/mL [169]
		Antimicrobial	*E. coli* (G-)	Active at 10 µg [169]
	434	Antimicrobial	*M. luteus* (G+)	Active at 5 µg [169]
		Antimicrobial	*B. subtilis* (G+)	Active at 5 µg [169]
		Antifungal	*P. oxalis*	8 mm at 5 µg [169]
		Antimicrobial	*M. luteus* (G+)	Active at 5 µg [169]
		Anticancer	Epidermoid carcinoma	ED_{50}: 2.4 µg/mL [169]
	455	Anticancer	Vinblastine resistant carcinoma	ED_{50}: 4.1 µg/mL [169]
		Anticancer	Human prostate cancer	ED_{50}: 12.9 µg/mL [169]
		Anticancer	Human breast cancer	ED_{50}: 12.9 µg/mL [169]
		Antimicrobial	*E. coli* (G-)	Active at 5 µg [169]
	458	Antimicrobial	*M. luteus* (G+)	Active at 5 µg [169]
		Antimicrobial	*B. subtilis* (G+)	Active at 5 µg [169]
		Antifungal	*P. oxalis*	16 mm at 5 µg [169]
		Antimicrobial	*E. coli* (G-)	Active at 5 µg [169]
		Antimicrobial	*M. luteus* (G+)	Active at 5 µg [169]
	456	Antimicrobial	*B. subtilis* (G+)	Active at 10 µg [169]
		Antifungal	*P. oxalis*	6 mm at 5 µg [169]
		Anticancer	Human prostate cancer	ED_{50}: 9.1 µg/mL [169]
		Anticancer	Human breast cancer	ED_{50}: 6.4 µg/mL [169]
		Antimicrobial	*M. luteus* (G+)	Active at 1 µg [169]
		Antifungal	*P. oxalis*	6 mm at 5 µg [169]
	459	Anticancer	Human epidermoid carcinoma	ED_{50}: 5.4 µg/mL [169]
		Anticancer	Vinblastine resistant carcinoma	ED_{50}: 10.7 µg/mL [169]
		Anticancer	Human prostate cancer	ED_{50}: 1.7 µg/mL [169]
		Anticancer	Human breast cancer	ED_{50}: 2.4 µg/mL [169]
		Antimicrobial	*M. luteus* (G+)	Active at 5 µg [169]
		Antimicrobial	*B. subtilis* (G+)	Active at 5 µg [169]
	461	Antifungal	*P. oxalis*	12 mm at 5 µg [169]
		Anticancer	Human epidermoid carcinoma	ED_{50}: 3.9 µg/mL [169]
		Anticancer	Vinblastine resistant carcinoma	ED_{50}: 5.6 µg/mL [169]
		Anticancer	Human prostate cancer	ED_{50}: 3.6 µg/mL [169]
		Anticancer	Human breast cancer	ED_{50}: 4.0 µg/mL [169]
		Antiplasmodia	*P. falciparum* (D6)	IC_{50}: 3.8 µg/mL [169]
		Antifungal	*P. oxalis*	8 mm at 5 µg [169]
		Anticancer	Human epidermoid carcinoma	ED50: 6.1 µg/mL [169]
	460	Anticancer	Vinblastine resistant carcinoma	ED50: 5.3 µg/mL [169]
		Anticancer	Human prostate cancer	ED50: 4.5 µg/mL [169]
		Anticancer	Human breast cancer	ED50: 3.8 µg/mL [169]
		Antimicrobial	*M. luteus* (G+)	Active at 5 µg [169]
		Antimicrobial	*B. subtilis* (G+)	Active at 5 µg [169]
	452	Antifungal	*P. oxalis*	14 mm at 5 µg [169]
		Anticancer	Human epidermoid carcinoma	ED_{50}: 4.1 µg/mL [169]
		Anticancer	Vinblastine resistant carcinoma	ED_{50}: 3.9 µg/mL [169]
		Anticancer	Human prostate cancer	ED_{50}: 3.6 µg/mL [169]
		Anticancer	Human breast cancer	ED_{50}: 2.8 µg/mL [169]

S33. Biological Activity Summary of Red Algae (Rhodophyta) (continued)

D. pulchra		Antimicrobial	*E. coli* (G-)	Active at 1 μg [169]
		Antimicrobial	*B. subtilis* (G+)	Very Active at 5 μg [169]
	462	Anticancer	Human epidermoid carcinoma	ED_{50}: 7.6 μg/mL [169]
		Anticancer	Vinblastine resistant carcinoma	ED_{50}: 10.5 μg/mL [169]
		Anticancer	Human prostate cancer	ED_{50}: 6.6 μg/mL [169]
		Anticancer	Human breast cancer	ED_{50}: 6.7 μg/mL [169]
		Antimicrobial	*E. coli* (G-)	Active at 10 μg [169]
		Antimicrobial	*M. luteus* (G+)	Very Active at 5 μg [169]
		Antimicrobial	*B. subtilis* (G+)	Very Active at 1 μg [169]
	435	Antifungal	*P. oxalis*	14 mm at 5 μg [169]
		Anticancer	Human lung cancer	ED_{50}: 1.7 μg/mL [169]
		Anticancer	Human epidermoid carcinoma	ED_{50}: 0.8 μg/mL [169]
		Anticancer	Vinblastine resistant carcinoma	ED_{50}: 2.1 μg/mL [169]
		Anticancer	Human prostate cancer	ED_{50}: 0.5 μg/mL [169]
		Anticancer	Human breast cancer	ED_{50}: 1.6 μg/mL [169]
		Antimicrobial	*M. luteus* (G+)	Very Active at 5 μg [169]
	443	Antimicrobial	*B. subtilis* (G+)	Very Active at 1 μg [169]
		Antifungal	*P. oxalis*	22 mm at 5 μg [169]
		Antimicrobial	*E. coli* (G-)	Very Active at 1 μg [169]
		Antimicrobial	*M. luteus* (G+)	Very Active at 1 μg [169]
		Antimicrobial	*B. subtilis* (G+)	Very Active at 1 μg [169]
		Antifungal	*P. oxalis*	26 mm at 5 μg [169]
	441	Anticancer	Human lung cancer	ED_{50}: 0.3 μg/mL [169]
		Anticancer	Human epidermoid carcinoma	ED_{50}: 0.4 μg/mL [169]
		Anticancer	Vinblastine resistant carcinoma	ED_{50}: 3.4 μg/mL [169]
		Anticancer	Human prostate cancer	ED_{50}: 0.2 μg/mL [169]
		Anticancer	Human breast cancer	ED_{50}: <0.1 μg/mL [169]
		Antimicrobial	*E. coli* (G-)	Very Active at 1 μg [169]
		Antimicrobial	*M. luteus* (G+)	Very Active at 1 μg [169]
		Antimicrobial	*B. subtilis* (G+)	Very Active at 1 μg [169]
	436	Antifungal	*P. oxalis*	31 mm at 5 μg [169]
		Anticancer	Human lung cancer	ED_{50}: 0.6 μg/mL [169]
		Anticancer	Human epidermoid carcinoma	ED_{50}: 0.5 μg/mL [169]
		Anticancer	Vinblastine resistant carcinoma	ED_{50}: 0.2 μg/mL [169]
		Anticancer	Human prostate cancer	ED_{50}: <0.1 μg/mL [169]
		Anticancer	Human breast cancer	ED_{50}: 0.3 μg/mL [169]
		Antimicrobial	*M. luteus* (G+)	Very Active at 5 μg [169]
		Antifungal	*P. oxalis*	3 mm at 5 μg [169]
		Anticancer	Human breast cancer	ED_{50}: 9.8 μg/mL [169]
	437	Anticancer	Human fibrosarcoma	ED_{50}: 2.2 μg/mL [169]
		Anticancer	Human melanoma	ED_{50}: 5.3 μg/mL [169]
		Anticancer	Mouse lymphoid neoplasm	ED_{50}: <5 μg/mL [169]
		Anticancer	Human prostate cancer	ED_{50}: 7.3 μg/mL [169]
		Anticancer	Human breast cancer	ED_{50}: 10.2 μg/mL [169]
		Anticancer	Human glioblastoma	ED_{50}: 10.0 μg/mL [169]
		Antimicrobial	*E. coli* (G-)	Active at 1 μg [169]
		Antimicrobial	*M. luteus* (G+)	Very Active at 5 μg [169]
		Antimicrobial	*B. subtilis* (G+)	Very Active at 5 μg [169]
	440	Antifungal	*P. oxalis*	18 mm at 5 μg [169]
		Anticancer	Human lung cancer	ED_{50}: 1.3 μg/mL [169]
		Anticancer	Human epidermoid carcinoma	ED_{50}: 0.4 μg/mL [169]
		Anticancer	Vinblastine resistant carcinoma	ED_{50}: 4.1 μg/mL [169]
		Anticancer	Human prostate cancer	ED_{50}: <0.1 μg/mL [169]
		Anticancer	Human breast cancer	ED_{50}: 0.3 μg/mL [169]
		Antiplasmodial	*P. falciparum* (D6)	IC_{50}: 6.8 μg/mL [169]

S33. Biological Activity Summary of Red Algae (Rhodophyta) (continued)

		Antimicrobial	*E. coli* (G-)	Active at 5 µg [169]
		Antimicrobial	*M. luteus* (G+)	Very Active at 5 µg [169]
		Antimicrobial	*B. subtilis* (G+)	Very Active at 1 µg [169]
		Antifungal	*P. oxalis*	21 mm at 5 µg [169]
	444	Anticancer	Human lung cancer	ED_{50}: 1.5 µg/mL [169]
		Anticancer	Human epidermoid carcinoma	ED_{50}: 0.1 µg/mL [169]
		Anticancer	Vinblastine resistant carcinoma	ED_{50}: 3.6 µg/mL [169]
		Anticancer	Human prostate cancer	ED_{50}: 2.0 µg/mL [169]
		Anticancer	Human breast cancer	ED_{50}: <0.1 µg/mL [169]
		Antiplasmodial	*P. falciparum* (D6)	IC_{50}: 4.6 µg/mL [169]
D. pulchra		Antimicrobial	*E. coli* (G-)	Very Active at 5 µg [169]
		Antimicrobial	*M. luteus* (G+)	Very Active at 5 µg [169]
		Antimicrobial	*B. subtilis* (G+)	Very Active at 1 µg [169]
		Antifungal	*P. oxalis*	18 mm at 5 µg [169]
	447	Anticancer	Human lung cancer	ED_{50}: 1.0 µg/mL [169]
		Anticancer	Human epidermoid carcinoma	ED_{50}: 0.4 µg/mL [169]
		Anticancer	Vinblastine resistant carcinoma	ED_{50}: 1.2 µg/mL [169]
		Anticancer	Human prostate cancer	ED_{50}: 3.0 µg/mL [169]
		Anticancer	Human breast cancer	ED_{50}: 0.4 µg/mL [169]
		Antiplasmodial	*P. falciparum* (D6)	IC_{50}: 4.6 µg/mL [169]
		Antimicrobial	*E. coli* (G-)	Active at 5 µg [169]
		Antimicrobial	*M. luteus* (G+)	Very Active at 1 µg [169]
		Antimicrobial	*B. subtilis* (G+)	Very Active at 1 µg [169]
	445	Antifungal	*P. oxalis*	23 mm at 5 µg [169]
		Anticancer	Human lung cancer	ED_{50}: 1.7 µg/mL [169]
		Anticancer	Human epidermoid carcinoma	ED_{50}: 0.5 µg/mL [169]
		Anticancer	Vinblastine resistant carcinoma	ED_{50}: 0.6 µg/mL [169]
		Anticancer	Human prostate cancer	ED_{50}: 6.7 µg/mL [169]
		Anticancer	Human breast cancer	ED_{50}: 0.3 µg/mL [169]
		Antiplasmodial	*P. falciparum* (D6)	IC_{50}: 2.8 µg/mL [169]
		Antimicrobial	*E. coli* (G-)	Active at 5 µg [169]
		Antimicrobial	*M. luteus* (G+)	Active at 5 µg [169]
		Antimicrobial	*B. subtilis* (G+)	Very Active at 1 µg [169]
	446	Antifungal	*P. oxalis*	14 mm at 5 µg [169]
		Anticancer	Human lung cancer	ED_{50}: 1.1 µg/mL [169]
		Anticancer	Human epidermoid carcinoma	ED_{50}: 0.3 µg/mL [169]
		Anticancer	Vinblastine resistant carcinoma	ED_{50}: 0.5 µg/mL [169]
		Anticancer	Human prostate cancer	ED_{50}: 2.1 µg/mL [169]
		Anticancer	Human breast cancer	ED_{50}: <0.1 µg/mL [169]
		Antiplasmodial	*P. falciparum* (D6)	IC_{50}: 2.2 µg/mL [169]
		Antimicrobial	*E. coli* (G-)	Active at 5 µg [169]
		Antimicrobial	*M. luteus* (G+)	Very Active at 5 µg [169]
		Antimicrobial	*B. subtilis* (G+)	Very Active at 5 µg [169]
		Antifungal	*P. oxalis*	13 mm at 5 µg [169]
		Anticancer	Human breast cancer	ED_{50}: 6.4 µg/mL [169]
	438	Anticancer	Human lung cancer	ED_{50}: 10.3 µg/mL [169]
		Anticancer	Human melanoma	ED_{50}: 4.2 µg/mL [169]
		Anticancer	Human colon cancer	ED_{50}: 12.4 µg/mL [169]
		Anticancer	Mouse lymphoid neoplasm	ED_{50}: >5 µg/mL [169]
		Anticancer	Human epidermoid carcinoma	ED_{50}: 10.5 µg/mL [169]
		Anticancer	Human prostate cancer	ED_{50}: 9.3 µg/mL [169]
		Anticancer	Human breast cancer	ED_{50}: 8.6 µg/mL [169]
L. elata	Pure Compounds:			
		Antitumour	HeLa	IC50: 1.3 µM [132]
		Antitumour	Hep-2	IC50: 2.0 µM [132]
		Antifouling	*Alteromonas* sp1.	25-30 mm at 30 µg/disc [134]
		Antifouling	*Alteromonas* sp2.	12-18 mm at 30 µg/disc [134]
	Elatol (378)	Antifouling	*Proteus mirabilis*	12-18 mm at 30 µg/disc [134]
		Antifouling	*Proteus* sp.	7-12 mm at 30 µg/disc [134]
		Antifouling	*Cytophaga-flavobacterium*	25-30 mm at 30 µg/disc [134]
		Antifouling	*Vibrio* sp.	25-30 mm at 30 µg/disc [134]
		Antibacterial	*S. aureus* (G+)	12-18 mm at 30 µg/disc [134]

S33. Biological Activity Summary of Red Algae (Rhodophyta) (continued)

L. elata	Pacifenol (**377**)	Cytotoxicity	Brine Shrimp Assay	Significant at 23 μg/mL [136]
	(**381**)	Cytotoxicity	Brine Shrimp Assay	Moderate at 23 μg/mL [136]
	Crude Extract:			
	MeOH:DCM	Antiviral	*Herpes simplex*, Polio	~ [131]
	3:1	Antitumour	P388	IC_{50}: 168,632 ng/mL [131]
		Antifungal	*Trichophyton mentagrophytes*	~ [131]
L. filiformis	Pure Compounds:			
	(**366** and **367**)	P-gp Inhibitor	P-glycoprotein	~ [126]
	Filiformin (**393**)	Antitumour	P388	IC_{50}: >43 μM [144]
	Filiforminol (**397**)	Antitumour	P388	IC_{50}: >40 μM [144]
	Allolaurinterol (**386**)	Antitumour	P388	IC_{50}: >43 μM [144]
	Bromolaurenisol (**390**)	Antitumour	P388	IC_{50}: >34 μM [144]
	(**392**)	Cytotoxicity	Brine shrimp mortality	90% mortality at 23 μg/mL [136]
	Pre-pacifinol	Antibacterial	*S. aureus* 12600 (G+)	10-100μg/mL full inhibition 48h[215]
	(**367**)	Antitumour	P388	IC50: 8.5 μg/mL [127]
	(**367**)	Antitumour	HeLa	IC50: 6.3 μg/mL [127]
	(**367**)	Antifeedant	*Haliotis discus hannai*	Ei: 0.88 at 75 μg [128]
	(**367**)	Antifeedant	*S. nudus*	Ei: 0.92 at 75 μg [128]
	(**367**)	Antifeedant	*S. intermedius*	Ei: 0.97 at 75 μg [128]
	(**366**)	Antitumour	Ehrlich carcinoma	90% inhibition at 50 μg/mL [129]
	(**366**)	Anthelmintic	*caliginosa*	significant at 10 % (w/v) [129]
	Pacifenol (**377**)	Cytotoxicity	Brine Shrimp Assay	Significant at 23 μg/mL [136]
	(**379**)	Cytotoxicity	Brine Shrimp Assay	Moderate at 23 μg/mL [136]
	(**380**)	Cytotoxicity	Brine Shrimp Assay	Moderate at 23 μg/mL [136]
	(**381**)	Cytotoxicity	Brine Shrimp Assay	Moderate at 23 μg/mL [136]
	(**390**)	Cytotoxicity	NSCLC-N6	IC50: 26.5 μM [145]
	(**386**)	Antibacterial	*S. aureus* (G+)	2xMIC: 6.25 μg/mL [146]
	(**386**)	Antibacterial	VCM E. faecium	moderate [146]
	(**386**)	Antitubercular	*M. tuberculosis*	moderate [147]
	(**401**)	Antitumour	P388	T/C 175 at 400 mg/kg [151]
	(**405**)	Antifeedant	*X. helleri* (Swordtail fish)	Significant [161]
	Crude Extracts:			
		Antiviral	*H. simplex*, Polio	Moderate [144]
	MeOH:DCM	Antibacterial	*B. subtilis* (G+)	Moderate [144]
	3:1	Antifungal	*T. mentagrophytes*	Moderate [144]
P. angustum	Pure Compounds:			
	Plocamenone (**352**)	Antitumour	P388	IC_{50}: 157.5 ng/mL [120]
	Plocamenone (**352**)	Antitumour	P388	IC_{50}: <97.5 ng/mL [120]
	Plocamenone (**352**)	Antitumour	P388	IC_{50}: <97.5 ng/mL [120]
	Mixture:			
	Plocamenone (**352**) + isoplocamenone (**353**)	Antitumour	P388	IC_{50}: <97.5 ng/mL [120]
	Crude Extracts:			
		Antitumour	P388	IC_{50}: <4875 ng/mL [120]
		Antitumour	P388	IC_{50}: <4875 ng/mL [120]
	MeOH:DCM	Antitumour	P388	IC_{50}: 22,743 ng/mL [120]
	3:1	Antiviral	*H. simplex*, Polio	~ [120]
		Antibacterial	*B. subtilis* (G+)	15 mm at 50 mg/mL [120]
		Antifungal	*C. albicans*	10 mm at 50 mg/mL [120]
		Antifungal	*T. mentagrophytes*	10 mm at 50 mg/mL [120]
		Antifungal	*C. resinae*	20 mm at 50 mg/mL [120]
P. australasica				
	Pure Compounds:			
	**425**	Cytotoxic	PC3 cells	IC_{50}: 0.44 μM [166]
	**490**	Cytotoxic	PC3 cells	IC_{50}: 10.0 μM [166]
P. leptophyllum	Pure Compounds:			
		Antifeedant	*T. cornutus*	Active at 40 μg [124]
	**365**	Antifeedant	*O. pfeifferi*	Active at 40 μg [124]
		Antifeedant	*H. discus*	Active at 40 μg [124]
		Antifeedant	*S. intermedius*	Active at 40 μg [124]

S33. Biological Activity Summary of Red Algae (Rhodophyta) (continued)

P. mertensii	Pure Compounds:			
			Spodoptera frugiperda	94% mortality at 1000 ppm [117]
		Antifeedant and	*Diabrotica undecimpunctata*	100% mortality at 1000 ppm [117]
	(**342**)	Insecticidal	*Tetranychus urticae*	100% mortality at 1000 ppm [117]
			Alphis fabae	100% mortality at 1000 ppm [117]
		Antifungal	Apple scab	72% mortality at 100 ppm [117]
		Antifungal	Broad Bean botryitis	73% mortality at 100 ppm [117]
		Antifeedant and	*Spodoptera frugiperda*	40% mortality at 1000 ppm [117]
	Mertensene (**340**)	Insecticidal	*Alphis fabae*	79% mortality at 1000 ppm [117]
		Antifungal	*Tetranychus urticae*	42% mortality at 1000 ppm [117]
			Grape downy mildew	59% mortality at 100 ppm [117]
	Crude Extracts:			
	MeOH:DCM	Antitumour	P388	IC_{50}: 254,448 ng/mL [116]
	3:1	Antiviral	*H. simplex*	Slight [116]
		Antifungal	*T. mentagrophytes*	Slight [116]
R. membranacea	Pure Compounds:			
	409	Anticancer	HL-60	IC_{50}: 38 μM [163]
	413	Anticancer	HL-60	IC_{50}: 78 μM [163]
	415	Anticancer	HL-60	IC_{50}: 61 μM [163]
	410	Anticancer	HL-60	IC_{50}: 49 μM [163]
	421	Anticancer	HL-60	IC_{50}: 28 μM [163]
	424	Anticancer	HL-60	IC_{50}: 61 μM [163]
	409	Antifungal	*S. cerevisiae*	IC_{50}: 61 μM [163]
	413	Antifungal	*S. cerevisiae*	IC_{50}: 61 μM [163]
	415	Antifungal	*S. cerevisiae*	IC_{50}: 61 μM [163]
	410	Antifungal	*S. cerevisiae*	IC_{50}: 61 μM [163]
	421	Antifungal	*S. cerevisiae*	IC_{50}: 61 μM [163]
	424	Antifungal	*S. cerevisiae*	IC_{50}: 61 μM [163]
